# Identification of genetic variations associated with epsilon-poly-lysine biosynthesis in *Streptomyces albulus ZPM* by genome sequencing

**DOI:** 10.1038/srep09201

**Published:** 2015-03-17

**Authors:** Lin Wang, Chunhui Gao, Nan Tang, Songnian Hu, Qingfa Wu

**Affiliations:** 1 School of Life Sciences, University of Science & Technology of China, Hefei, Anhui 230027, China; 2 CAS Key Laboratory of Innate Immunity and Chronic Disease, School of Life Sciences, University of Science & Technology of China, Hefei, Anhui 230027, China; 3 CAS Key Laboratory of Genome Sciences & Information, Beijing institute of Genomics, CAS, 100101

## Abstract

The biosynthesis of the antibiotic epsilon-poly-lysine (ε-PL) in *Streptomyces albulus* is performed by polylysine synthase (*pls*); however, the regulatory mechanism of this process is still unknown. Here, we first obtained the complete genome sequence of *S. albulus* ZPM, which consists of 9,784,577 bp and has a GC content of 72.2%. The genome houses 44 gene clusters for secondary metabolite biosynthesis, in which 20 gene clusters are involved in the biosynthesis of polyketides and nonribosomally synthesized peptides. High-throughput sequencing was further performed, and genetic variants were identified from pooled libraries consisting of the 30 highest-yield mutants or 30 lowest-yield mutants. More than 350 genetic variants associated with ε-PL yield have been identified. One hundred sixty-two affected proteins, from important metabolic enzymes to novel transcriptional regulators, were identified as being related to ε-PL synthesis. HrdD, one of the affected genes, is a sigma factor that shows the most sensitive response to pH change and contains a non-synonymous mutation (A132V) in mutant strains with lower ε-PL yields. Electrophoretic mobility shift assays showed that the *pls* gene is likely regulated by transcriptional activator HrdD. The data obtained in this study will facilitate future studies on ε-PL yield improvement and industrial bioprocess optimization.

S*treptomyces* species are Gram-positive bacteria that predominantly inhabit highly complex and competitive soil environments. The secondary metabolites produced by *Streptomyces* species have been the sources of antibiotics, parasiticides, herbicides and pharmacologically active substances^1^. Currently, more than half of the medically important antimicrobial and antitumor agents are provided by *Streptomyces* species^2^. ε-poly-lysine (ε-PL) is a homo-poly-amino acid characterized by the peptide backbone between the carboxyl and ε-amino groups of L-lysine^3^. ε-PL was first isolated from *Streptomyces albulus* and characterized as a novel peptide antibiotic in the 1970s^4^. ε-PLs with chain lengths larger than nine L-lysine residues exhibit antimicrobial activities against a wide spectrum of microorganisms, including gram-positive and gram-negative bacteria and phages^3^,^5^,^6^. As ε-PL is safe, biodegradable, and stable at high temperature and low-pH environments^3^, it has been introduced as a food preservative in many countries^3^. The application of ε-PL in antiobesity and selective endotoxin-removal action has been reported^7^. In addition to *S. albulus*, ε-PL has also been isolated from *S. griseofuscus*^8^, *Streptomyces sp*. M-Z18^9^, *Streptomyces sp*. GIM8^10^ and *Kitasatosporasp* PL6-3^11^.

The synthesis of ε-PL in *S.* *albulus* is controlled by an unusual non-ribosomal peptide synthase (NRPS), poly-lysine synthase (Pls)^12^. When the *pls* gene is disrupted, *S. albulus* loses its capacity to produce ε-PL^12^. Consistently, the NRPS Pls is able to utilize L-lysine as a substrate to synthesize ε-PL *in vitro*^12^. Additionally, ε-PL can be degraded into lysine monomers by the proteases Pld and PldII in *S. albulus*^5^,^13^. Interestingly, *pldII*, which encodes the main degradase in *S. albulus*, is located adjacent to the *pls* gene, with a 4-bp overlap, and they form a single operon in the genome. The fermentation of *S. albulus* in media containing only glucose and ammonium sulfate, as carbon and nitrogen sources, respectively, results in ε-PL production^3^. Therefore, the lysine biosynthesis pathway is essential because L-aspartic acid is the initial building block of ε-PL *in vivo*. It is noteworthy that *pls* is not a constitutive gene but is induced ~12 hours after fermentation, when the pH of the medium is lower than 5^13^, which implies that *pls* expression is strictly regulated and low pH may be a possible inducer. The success of pH control strategies on improving PL yield^14^ has also implied that pH may have a regulatory function in ε-PL synthesis. Although the biosynthesis and degradation mechanisms of ε-PL have been elucidated, the regulatory mechanism of ε-PL synthesis, particularly *in vivo*, is still unclear.

In the current study, we first performed whole-genome shotgun sequencing of *S. albulus* ZPM, which was isolated from the soil and produces the homopolymer antibiotic, ε-PL. Through genome annotation, we characterized a large portion of transcriptional regulators and many secondary metabolite gene clusters. Furthermore, pan-genome analysis revealed that strain-specific genes are highly abundant in *S. albulus* ZPM compared with other *Streptomycetes* species. To study the mechanism of the regulation of ε-PL yield, a large mutant library of strains was constructed using both physical and chemical mutagenesis strategies. The genomic DNAs (gDNAs) of the high- and low-yield groups were pooled, and high-throughput DNA sequencing was performed. Single-nucleotide polymorphism (SNP) and Indel calling were subsequently performed, and 208 and 163 SNPs/Indels that were specific to the low- or high-yield groups, respectively, were identified. In total, the amino acid sequences of 162 proteins that were involved in diverse cellular metabolism processes were affected by these genetic variations. In conclusion, our findings enhance the understanding of ε-PL biosynthesis.

## Results

### Genome analysis of *Streptomyces albulus* ZPM

The strain *S. albulus* ZPM, which produces ε-PL, was isolated from the soil of Zi-Peng Mountain (ZPM) west of Hefei, China using the methylene blue screening method^15^ (Fig. S1A). The *pls* gene was amplified from the strain's gDNA by PCR and was verified by Sanger sequencing (Fig. S1B). The ε-PL yield of this strain in a shaking flask is ~0.79 g/L, which is 30% higher than the industrial *S. albulus* 410 strain (~0.6 g/L) under similar circumstances^14^. Phylogenetic analysis of 16S rRNA revealed that the strain was closest to *S. albulus*; thus, the stain was designated *S. albulus* ZPM (Fig. S1C).

**Figure 1:**
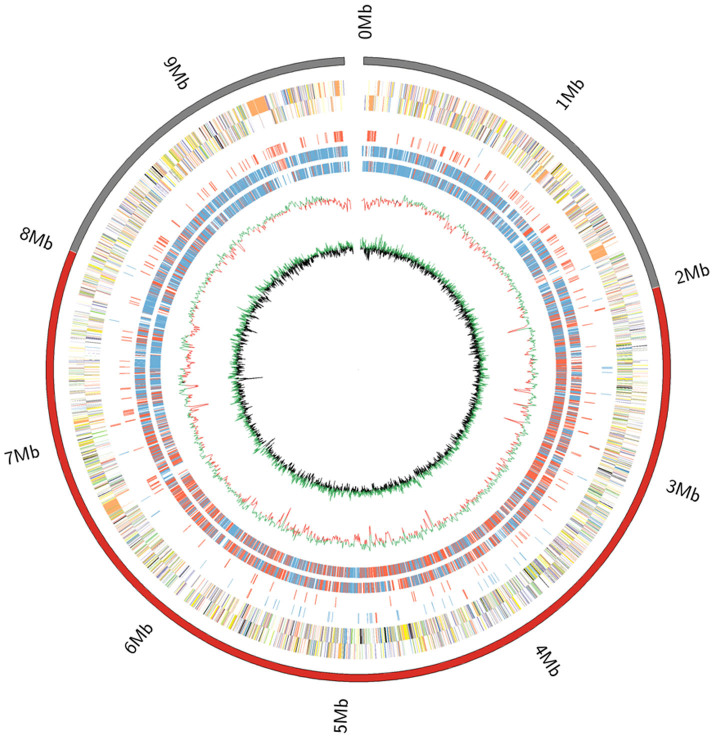
Circular representation of the *S. albulus* ZPM chromosome. Circles 1 and 2, all genes (forward and reverse strands, respectively) are color-coded by function (blue, RNA processing and modification; vlblue, Chromatin structure and dynamics; chrm, Energy production and conversion; churn, Cell cycle control, cell division, chromosome partitioning; lgreen, Amino acid transport and metabolism; vlgreen, Nucleotide transport and metabolism; grey, Carbohydrate transport and metabolism; dblue, Coenzyme transport and metabolism; dyellow, Translation, ribosomal structure and biogenesis; vlred, Transcription; vlyellow, Replication, recombination and repair; lpurple, Cell wall/membrane/envelope biogenesis; black, Posttranslational modification, protein turnover, chaperones; vlorange, Inorganic ion transport and metabolism; lorange, Secondary metabolite biosynthesis, transport and catabolism; dpurple, General function prediction only; vlpurple, Function unknown; lred, Signal transduction mechanisms; dgrey, Intracellular trafficking, secretion, and vesicular transport; vvlgrey, Defense mechanisms); Circle 3, tRNA (red) and rRNA operon (blue); Circle 4, secondary metabolism genes; Circles 5 and 6 (forward and reverse strands), distributions of conserved (red) and strain-specific genes (blue) in the *S. albulus* ZpM genome compared with 11 other *Streptomyces* species; Circle 7, GC content; Circle 8, GC bias ([G − C/G + C], green indicates values > 1, dark < 1). The inside scale is numbered clockwise in Mb. The outer scale indicates the core (red) and noncore (gray) chromosomal regions. The origin of replication (Ori) is also indicated.

The complete genome sequence of *S. albulus* ZPM revealed a single linear chromosome composed of 9,784,577 bp, with the pZPM234 plasmid and an average G + C content of 72.2%. The 36,995-bp plasmid is homologous to the pNO33 plasmid and contains 37 genes that possibly code for membrane proteins, exonucleases and metabolic enzymes, along with proteins involved in partitioning (Fig. S2). The chromosome has a 139,385-bp terminal inverted repeat (TIR) with a remarkably low GC content of 66.7% at both ends (1 ~ 139385, 9645193 ~ 9784577) (Fig. 1). In total, 9197 genes have been identified in the *S. albulus* ZPM chromosome, including 9073 protein-coding genes, 18 ribosomal genes in 6 operons in the order 16S-23S-5S and 70 tRNA genes (Table S1). A total of 5816 of the 9073 protein-coding genes (64.1%) have been classified into at least one Cluster of Orthologous Groups (COG) with known or putative function (Fig. S3). In the TIR region, 106 genes have been identified, including 10 genes that are related to recombination, 5 involved in defense and 17 genes involved in secondary metabolite biosynthesis. The replication origin, *oriC*, contains 18 DnaA box-like sequences and is located at the center of the chromosome (5114740–5116614 bp). The terminal protein (Tp) and telomere-associated protein (Tap), which are encoded by SAZ_8415 and SAZ_8416, respectively, are responsible for telomere replication and are 28.70% and 55.88% identical to the Tap and Tpg proteins of *S. griseus*, respectively^16^. The proteins have no detectable similarities with the conserved Tp and Tap proteins of other *Streptomyces* species, such as *S. coelicolor A3 (2)*^17^ and *S. avermitilis*^18^.

Comparative genomic analysis shows that the chromosomal region ranging from position 2.0 Mb to position of 8.0 Mb of *S. albulus* ZPM is conserved and shows significant synteny among *Streptomyces* species; thus, the 6-Mb chromosomal region is defined as the core region of *S. albulus* ZPM, whereas the chromosomal regions at both ends are referred to as the variable regions (Fig. 1, Fig. S4). The lengths of the core regions of *Streptomyces* species range from 5.0 M to 7.5 M, which is proportional to their chromosome lengths (Fig. S4). Compared with *S. albulus* ZPM, the chromosomal sequences around the *oriC* appear to have significant genomic synteny with other *Streptomyces* species, although numerous inversions have been observed around this region (Fig. S4).

### Pan-genome analysis reveals a large portion of specific genes in *S. albulus* ZPM

**Figure 2:**
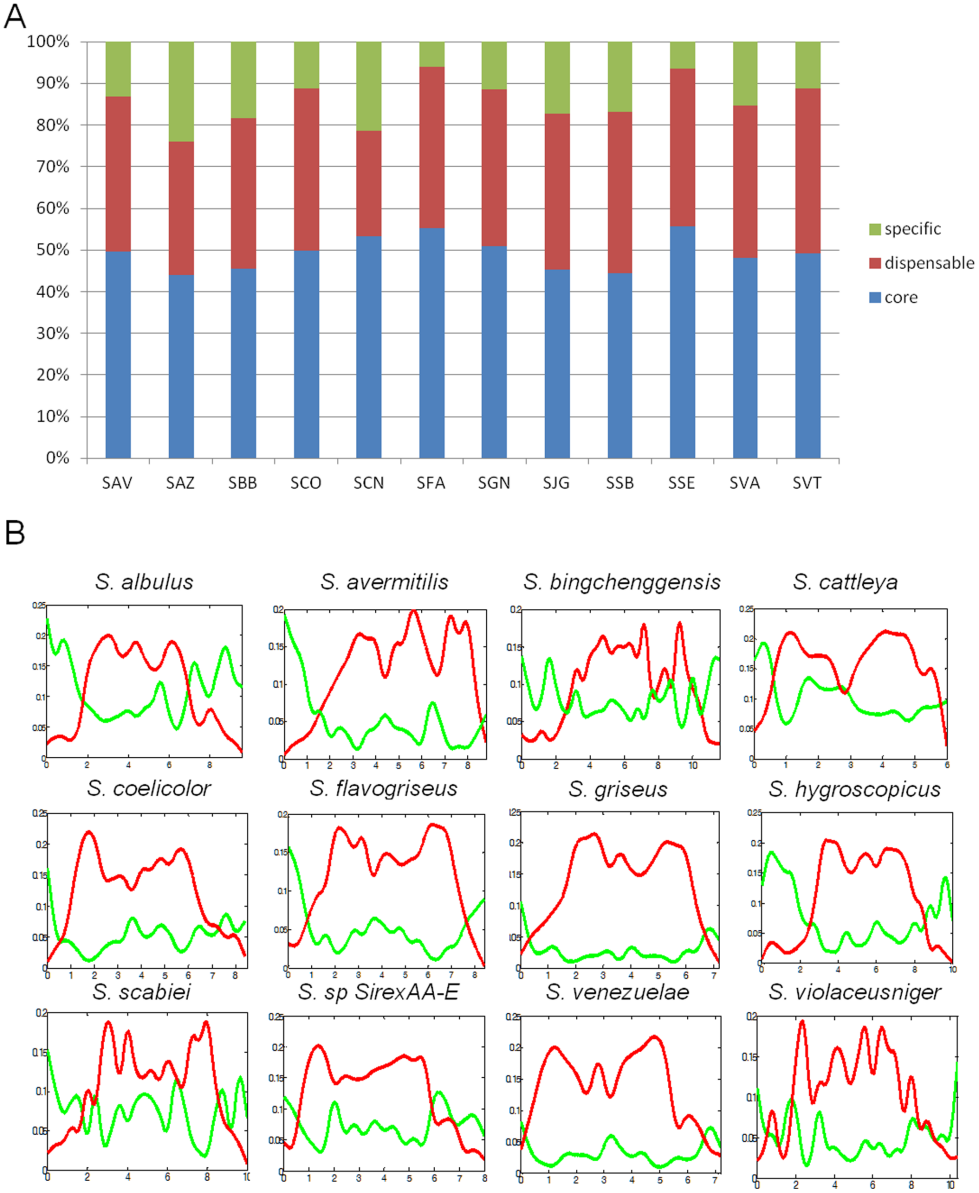
Pan-genome analysis among *Streptomyces* species. (A) Percentages of core (blue), dispensable (red) and specific (green) genes in *S. albulus* ZPM and 11 other *Streptomyces* species. The core genes represent genes shared by all 12 *Streptomyces* species, dispensable genes represent genes shared by at least two *Streptomyces* species, specific genes represent genes unique to one *Streptomyces* species. The 12 *Streptomyces* species are *S. avermitilis* (SAV), *S. albulus* ZPM (SAZ), *S. bingchenggensis* (SBB), *S. cattleya* (SCN), *S. coelicolor* (SCO), *S. flavogriseus* (SFA), *S. griseus* (SGN), *S. hygroscopicus* (SJG), *S. scabiei* (SSB), *S. sp. SirexAA-E* (SSE), *S. venezuelae* (SVA) and *S. violaceusniger* (SVT). (B) Density plot of the core (red) and strain-specific genes (green) along the chromosomes of twelve *Streptomyces* species. The bin size is 400 kb. The x- and y-axes represent the percentage of the chromosomal length and the proportion of genes in each *Streptomyces* species, respectively.

Pan-genome analyses were performed to analyze the core, lineage- and strain-specific genes of *S. albulus* ZPM together with those of 11 other *Streptomyces* species with complete genomes. First, an all-against-all BLASTP search with a cutoff E-value of 1e-5 was performed using sequences of proteins encoded in the genomes. Second, proteins with sequence identity > 50% and alignment coverage >70% were clustered; 93,586 proteins were grouped into 21,245 distinct protein families. The core families shared by the 11 *Streptomyces* species consisted of 1980 families, in which 43.92–55.60% of the genes of each species were included (Fig. 2A, Table S2). Although the numbers of core genes of the species appeared to be significantly different, the correlation between the core gene count and genome size was very high (R^2^ = 0.94, p-value = 1.73e-7, Table S2). The lowest and highest expansion rates of the core genes were 1.56 (3105/1980) for *S. cattleya* and 2.30 (4555/1980) for *S. bingchenggensis*, respectively, and the differences were also due to distinct genome sizes and gene counts (Table S2). Based on the phylogenetic relationships of the 12 *Streptomyces* species, lineage-specific genes (LSGs) shared by at least two clades occupied 25.20–39.58% of the genes in each species (Fig. S5). These LSGs contribute to species diversity and confer selective advantages, such as adaptation to different niches and antibiotic resistance. Similarly, the number of LSGs in each species showed strong correlations with the genome size (R^2^ = 0.87, p-value = 1.06e-5, Table S2).

Notably, the correlation between the number of specific genes in each species and the genome sizes of 12 *Streptomyces* species was 0.64, which was lower than that of the core and lineage-specific genes. Interestingly, 2172 of the specific genes were exclusively identified in *S. albulus* ZPM, which is the highest specific gene number among the 12 *Streptomyces* species. The ratio between the specific genes and the core genes in *S. albulus* ZPM is 0.545 (2172/3985), which is even higher than 0.40 (1840/4555) for *S. bingchenggensis*, which has a 22% larger genome (Fig. 2A, Table S2). Because the specific genes encode proteins involved in supplementary biochemical pathways and proteins whose functions contribute to unique phenotypic traits or adaptation to living niches, the high genome plasticity of *S. albulus* ZPM suggests that the bacteria have a higher competence for acquiring and adapting new genes in its genome. Density plots show that the specific genes are predominantly located in the variable genome (Fig. 2B). Correspondingly, 66.23% of the specific genes of *S. albulus* ZPM have been mapped to the ends of the chromosome. Gene ontology (GO) enrichment analysis of the specific genes of *S. albulus* ZPM revealed that the specific genes were rich in classes of phosphopantetheinyl transferases (GO: 0016740), DNA transposases (GO: 0004803) (FDR < 2.4e-4) and catalytic activity (GO: 0003824) at the molecular function level (Fig. S6). Because phosphopantetheine is an essential prosthetic group for polyketide synthase, this result suggests that *S. albulus* ZPM might have many specific PKS pathways. Meanwhile, the enrichment of proteins with DNA transposase activity may explain how *S. albulus* ZPM easily acquires and adapts other genes in its genome.

### Gene clusters and the potential for the production of secondary metabolites

*Streptomyces* are well-known producers of a variety of secondary metabolites, such as antibiotics, antiparasitics, and anticancer agents. Bioinformatics analyses revealed 44 gene clusters for secondary metabolites in *S. albulus* ZPM (Table 1), which was almost twice the 25 gene clusters of ***S. coelicolor* A3 (2)**. The total length of these gene clusters is 1,983,954 bp and accounts for 20% of the *S. albulus* ZPM genome. The distribution of those gene clusters on the chromosome is not uniform: 31 of the 44 gene clusters are located in the variable genome, which indicates that these gene clusters might have been horizontally acquired through evolution. The other 13 gene clusters are in the core region, which contains most of the essential genes.

Polyketide synthases (PKSs) and NRPSs are key players in the synthesis of secondary metabolites from primary metabolites. PKSs generate polyketide chains through the oligomerization of small carboxylic acids, whereas NRPSs utilize amino acids as building blocks to form amide or ester bonds. *S. albulus* ZPM includes 9 PKS clusters, 6 NRPS clusters and 5 hybrid PKS-NRPS clusters (Table 1). Of the 9 PKS gene clusters, *S. albulus* ZPM contains 4 gene clusters for type I PKSs, 2 gene clusters for type II PKSs, 1 gene cluster for type III PKSs and 1 gene cluster for type I and type IV hybrid PKSs. The *pls* NRPS gene cluster (PGC) is 42.7 kb in length and consists of 42 proteins. In PGC, the poly-lysine synthase encoded by SAZ_7727 and poly-lysine degradase II encoded by SAZ_7728 form an operon and overlap each other by 4 nt, indicating that translation of the two genes is coordinately regulated. In addition to the 20 PKS/NRPS clusters and 8 clusters of unknown function, the remaining 16 clusters have been predicted to direct the synthesis of secondary metabolites, including terpene, siderophores, lantipeptides and butyrolactone. One of the five lantipeptide gene clusters (cluster 27) of *S. albulus* ZPM is homologous to a gene cluster (class III lantipeptides) of *S. coelicolor* A3 (2). The genes of gene cluster 27 of *S. albulus* ZPM and of Class III lantipeptides of *S. coelicolor* A3 (2) are located in an unusually low-GC-content region that houses two transposase proteins (SAZ_6981, SAZ_7026) of *S. albulus* ZPM and one transposase protein (SCO6910) of *S. coelicolor A3* (2), respectively, which are essential for horizontal gene transfer (HGT) from other species.

### Identification of ε-PL yield-related genetic variants

To identify other genes involved in ε-PL biosynthesis, forward genetics was employed. *S. albulus* ZPM was used as a starting strain to perform mutagenic breeding using ultraviolet (UV) irradiation and nitrosoguanidine (NTG). One hundred eighty mutant strains with ε-PL yields from 0 g/L to 1.05 g/L were obtained from the original isolate of *S. albulus* ZPM. More than 70 of the strains had higher ε-PL yields than the original strain, indicating that ε-PL biosynthesis was enhanced by the mutated genes, whereas strains with lower ε-PL yields suggested that the biosynthesis pathway was impaired or even completely blocked by the mutated genes.

**Figure 3:**
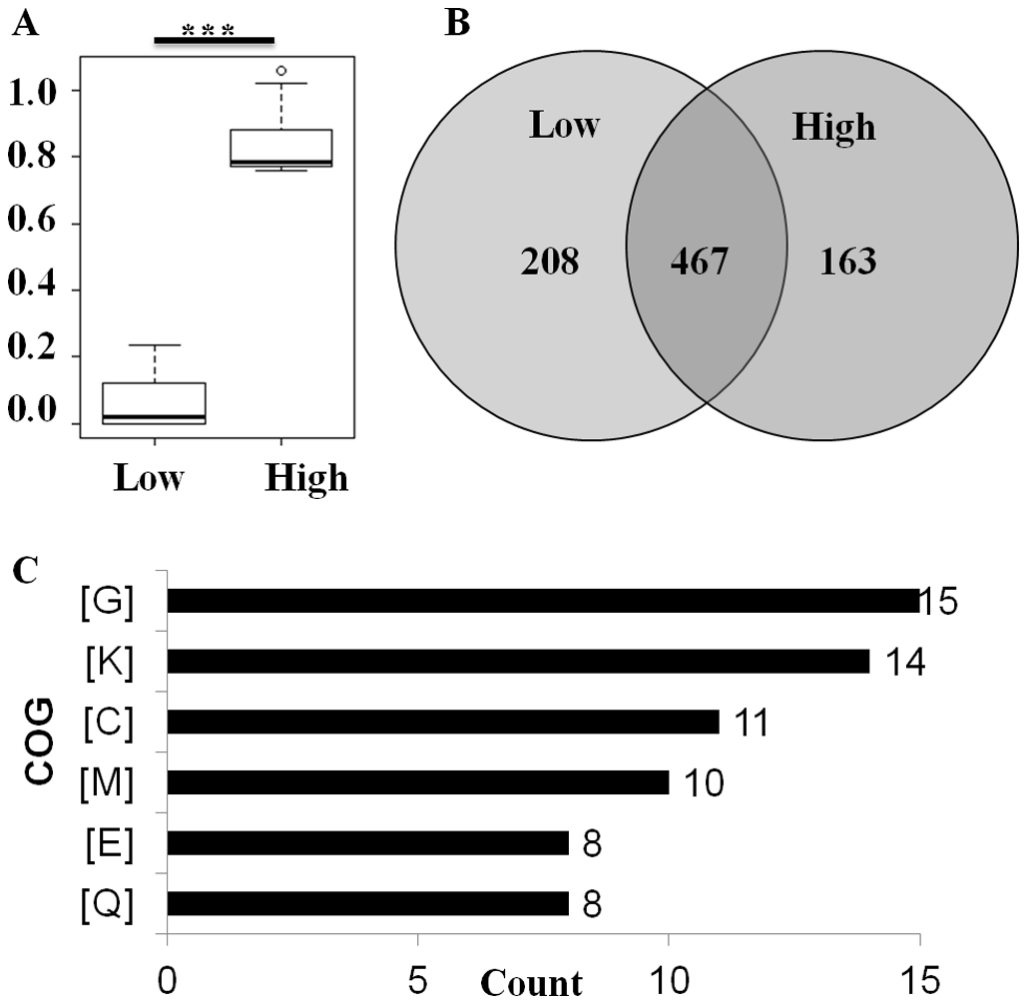
Identification and analysis of ε-PL yield-related genetic variants. (A) Box plot of the PL yield in the group-L and group-H mutant strains. Significance between the two groups is indicated by asterisks. (B) Venn diagram showing the genetic variants identified from the Low- and High-groups. (C) COG classifications of genes with non-synonymous mutations. [G]: Carbohydrate transport and metabolism; [K]: Transcription; [C]: Energy production and conversion; [M]: Cell wall/membrane/envelope biogenesis; [E]: Amino acid transport and metabolism; and [Q]: Secondary metabolites biosynthesis, transport and catabolism.

To determine the genetic variations related to ε-PL yield, equal amounts of the gDNAs of 30 mutants with the lowest ε-PL yields (group-L) and 30 mutants with the highest ε-PL yields (group-H) were pooled and sequenced using the Illumina HiSeq-2000 platform (Figure 3A, Table S3). In total, 675 and 630 genetic variants (SNP or Indel) were identified in group-L and group-H, respectively. Group-L and group-H share 467 common variants, accounting for 69.2% and 74.1% of the genomes of group-L and group-H, respectively (Table S4). To make the analysis simpler, only 208 group-L-specific variants and 163 group-H-specific variants that were believed to be related to ε-PL yield were analyzed further (Figure 3B).

For the group-L-specific genetic variants, 94 of 208 (45.2.0%) specific genetic variants were located in the coding region and led to amino acid changes, including 86 non-synonymous SNPs, 5 frameshift deletions/insertions, 2 non-frameshift deletions/insertions, and 1 stop gain/loss (Table 2). The group-H-specific genetic variations had similar distribution. Eighty-four of 163 (51.5%) group-H specific genetic variations were located in the coding regions and led to non-synonymous substitutions. Eighty-two group-L-specific and 52 group-H-specific SNPs/Indels mapped within 300 bp upstream or downstream of the coding regions of genes, which means that these SNPs/Indels might play a role in regulating the transcription of neighboring genes (Table 2). When combining the group-L- and group-H-specific genetic variants, 162 genes had non-synonymous substitutions.

### ε-PL yield is associated with both primary and secondary metabolism

COG analysis revealed that 66 of the 162 mutated genes could be assigned to at least one COG; in particular, genes from groups related to carbohydrate transport and metabolism, transcription and energy production were significantly enriched (Fig. 3C). In *Streptomycetes*, primary metabolism significantly influences secondary metabolism by providing precursors and reducing equivalents^21^. Although L-lysine is the substrate for ε-PL biosynthesis, these results show that ε-PL biosynthesis is not only related to the biosynthesis of L-lysine and ATP generation but is also associated with multiple cellular processes (Table S5).

Carbohydrate transport and metabolism is an essential component of central metabolism. It not only produces precursor metabolites for macromolecule biosynthesis but also generates ATP and other co-factors. In *S. albulus* ZPM, L-lysine is biosynthesized through the succinyl-diaminopimelic acid (DAP) pathway starting from L-aspartate. In addition to L-aspartate, other substrates, including pyruvate, succinyl-CoA, glutamate, ATP and NADPH are also required to complete the entire L-lysine biosynthesis pathway. L-aspartate is generated from oxaloacetate by the transfer of the amine group of L-glutamate to the keto group of oxaloacetate, whereas transamination of α-ketoglutarate results in glutamate. Pyruvate, α-ketoglutarate and oxaloacetate are key components of cellular metabolism, due to their contributions as substrates or intermediates in the fundamental processes of glycolysis, gluconeogenesis, and the citric acid cycle. Pyruvate kinase is a key enzyme in glycolysis that catalyzes the transfer of a phosphate group from phosphoenolpyruvate (PEP) to ADP to yield one molecule of pyruvate and one molecule of ATP. In group-L, an insertion mutation (261_262insCC) in pyruvate kinase (SAZ_6202) causes frameshifting of translation and impairments in the glycolytic pathway and L-lysine biosynthesis, which confirms that the ε-PL yield is correlated with pyruvate production and the downstream tricarboxylic acid cycle (TCA cycle). Non-synonymous SNPs have been identified in other genes involved in glycolysis and the TCA cycle, such as citrate synthase (N357H of SAZ_1229), NAD-glutamate dehydrogenase (P1341T of SAZ_3874), acetyl-CoA acetyltransferase (H185P of SAZ_8129) and 4-hydroxy-2-oxovalerate aldolase (T118P of SAZ_8139). Although the enzymatic activities of these mutated genes could not be determined, we suspect that these mutants most likely perturb the metabolic flux from glucose to L-lysine following the TCA cycle route.

Due to limitations in energy and/or the primary metabolite supply, the synthetic pathways of different secondary metabolites use common primary metabolites as precursors and compete with each other. The precursor L-lysine for ε-PL biosynthesis could be *de novo* synthesized from L-aspartate or transported from the surrounding environment. In our analysis, 21 mutated transporter genes were identified, including 8 ABC transporters, 6 transporters of the major facilitator superfamily, 6 other transporters and 1 potassium/proton antiporter. These proteins do not transport L-lysine directly but are responsible for content exchange between the extracellular environment and the cytoplasm, which is important for maintaining the stable physiological status of *S. albulus* ZPM and most likely indirectly impacts the ε-PL yield. L-aspartate is the precursor to several amino acids, including lysine, methionine, threonine, and isoleucine. No SNP has been identified in aspartokinase (SAZ_5045), which is the key enzyme of the lysine biosynthesis pathway. However, methionine synthase (SAZ_8322) with an R203M substitution, which is involved in the superpathway of lysine, threonine and methionine biosynthesis and catalyzes the formation of L-methionine from L-homocysteine, has been identified in group-L. Interestingly, non-synonymous substitutions have been identified in 2 polyketide synthase genes (SAZ_8963, SAZ_8662) and 1 NRPS-type-I PKS fusion protein (SAZ_1118); in these cases, oligomerization of small carboxylic acids catalyzed by these synthases might be disturbed, resulting in perturbation of the ε-PL yield through metabolic networks.

### Transcriptional activator HrdD binds with the promoter of *pls* gene *in vitro*

**Figure 4:**
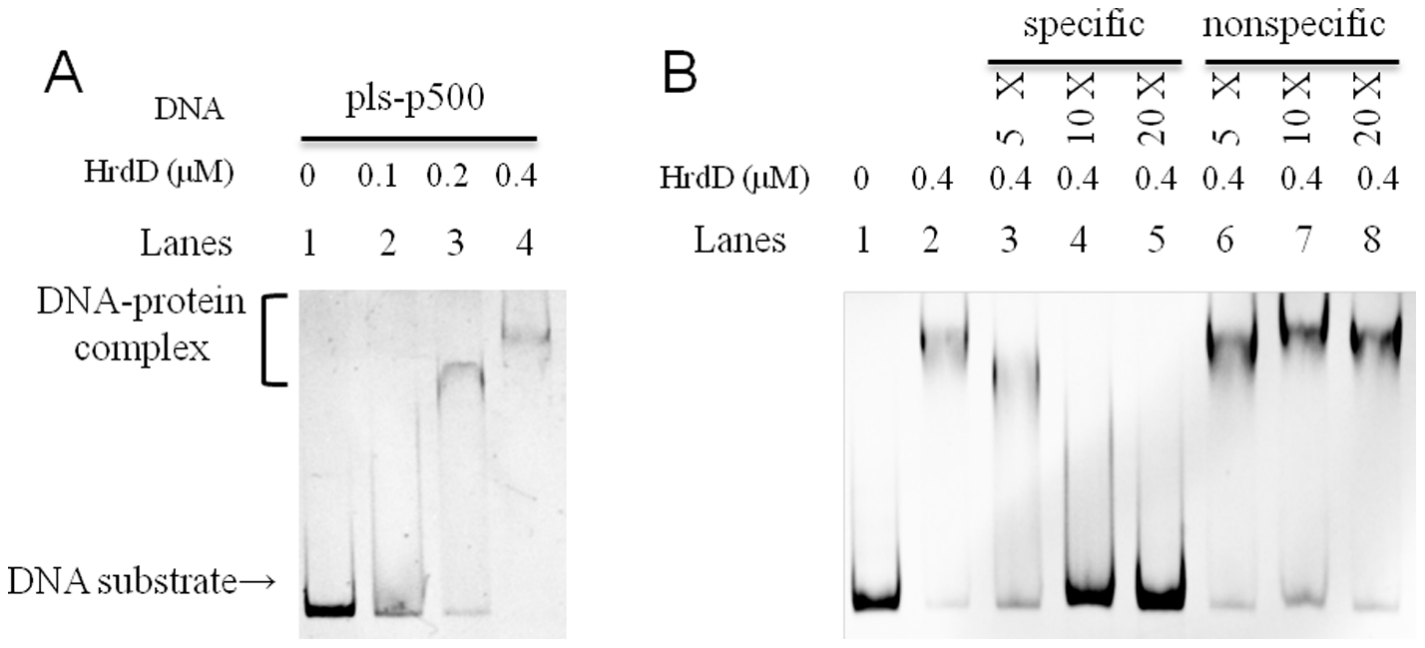
(A) EMSA assays of HrdD and the putative promoter of the *pls* gene. The recombinant HrdD protein was co-incubated with 30 nM 500-bp (lanes 1–4) upstream promoter DNA of the *pls* gene and were assayed on a 5% native PAGE gel. The final concentrations of the His-tag HrdD proteins in each mixture were 0, 0.1, 0.2 and 0.4 μM, as indicated in the second line. (B) In the competition assay, 3 nM of DNA substrates corresponding to the putative promoter of the *pls* gene (pls-p500) were labeled with fluorescein isothiocyanate (FITC) and were incubated with 0.4 μM his-tagged HrdD protein to compete with either excessive unlabeled pls-p500 DNA substrates or excessive nonspecific DNA substrates (lanes 7–9) derived from the gene coding green fluorescent protein(lanes 10–12).

Because ε-PL biosynthesis is induced by the acid condition (pH < 5) in *S. albulus* ZPM, we suspected that a sigma factor (σ factor) that responds to environmental pH change will guide RNA polymerase to bind the promoter and to initiate transcription of the *pls* gene. HrdD, encoded by SAZ_4132, is a sigma factor that shows the most sensitive response to pH change^19^ and contains a non-synonymous mutation (A132V) in mutant strains with lower ε-PL yields. To test whether HrdD binds to the promoter of the *pls* gene, electrophoretic mobility shift assays (EMSAs) were performed. 3 nM of DNA fragments of 500 bp that corresponded to the *pls* promoter (pls-p500) were incubated with his-tagged HrdD protein, and clear band shifts were observed when the concentration of HrdD was higher than 0.2 μM (Fig. 4A, lanes 3–4). To exclude the possibility of unspecific binding between HrdD to pls-p500, competition assay was done by incubating his-tagged HrdD with pls-p500 DNA fragments labeled with fluorescein isothiocyanate (FITC) and also with unlabeled cold pls-p500 DNA substrates (specific DNA) or the DNA fragments of green fluorescent protein gene (nonspecific DNA substrates) (Fig. 4B). The unlabeled cold pls-p500 DNA substrates competitively inhibited the binding of HrdD to the FITC-labeled pls-p500 DNA substrates (Fig. 4B, lanes 7–9). However, the non-specific DNA fragments, even up to 20 times excessive DNA substrates, have negligible effect on the binding of HrdD to the labeled pls-p500 DNA substrates (Fig. 4B, lanes 10–12). The competition assay definitively proved that HrdD is able to specifically bind to the promoter of *pls* gene and might monitor the *pls* expression and initiates ε-PL biosynthesis in response to pH changes *in vivo*.

## Discussion

In the past decades, much effort has been made to improve ε-PL yield, including genome shuffling and chemical mutagenesis. Although some industrial strains with higher yields have been obtained, the underlying regulatory mechanism of ε-PL biosynthesis is still unknown. In this study, we report the genome sequence of *S. albulus ZPM*, which is the first complete genome sequence of *S. albulus* without gaps or ambiguous regions. Currently, the genome sequences of two other *S. albulus* strains, *S. albulus CCRC 11814*^20^ and *S. albulus PD-1*^21^, not only contain contaminants from other species but also miss tens of thousands of nucleotides (data not shown). The high-quality complete sequence of *S. albulus ZPM* allows us to perform comparative genomic analysis of Streptomycetes species and identify genes associated with ε-PL biosynthesis by genome sequencing of *S. albulus ZPM* mutants.

Genome analysis showed that 162 proteins, from important metabolic enzymes to novel transcriptional regulators, were identified as related to ε-PL synthesis, which suggests that ε-PL biosynthesis is not only dependent on the supply of the primary metabolites L-lysine and ATP but also on multiple cellular processes. Meanwhile, our study shows at least 14 non-synonymous substitutions in the genes of transcription factors, including RNA polymerase alpha subunit A (SAZ_4270), the principal sigma factor HrdD (SAZ_4132) and the ECF subfamily RNA polymerase sigma factor (SAZ_4218, SAZ_5788). Previous studies have shown that the secondary metabolite yields of industrial strains could be enhanced by global transcription machinery engineering (gTME). For example, the application of gTME to *Saccharomyces cerevisiae* improved glucose/ethanol tolerance and increased biofuel production^22^,^23^. Thus, the transcription factors identified in this study might impact ε-PL yield in a similar manner.

## Methods

### Enzymes, plasmids and reagents

Restriction enzymes, DNA polymerase, dNTPs and all antibiotics were purchased from TaKaRa Biotech. PCR primers were synthesized by Sangon Biotech Company. All other reagents were purchased from Sigma unless specified.

### Strains and media

The original *S. albulus* ZPM strain was isolated from the soil of Zi-Peng Mountain, Hefei, China. The spore suspension of *S. albulus* was treated with UV (20 W) irradiation for 30–65 seconds at a 20-cm distance, followed with 0.5 g/L NTG for 30 minutes at 30°C and 180 rpm. All strains of *S. albulus* were cultured on SGB agar plates or in M_3_G broth at 30°C. *E. coli* strains were cultured in LB medium.

### Genome sequencing, assembly, annotation and analysis

gDNA was extracted using the UltraClean® Microbial RNA Isolation Kit following the manual, and Multiplex Identifier (MID)-tagged paired-end and mate-pair libraries were generated using the NextFlex DNA-seq preparation kit following the manufacturer's instructions. The libraries were pooled, and paired-end sequencing (2 × 100 bp) was performed using a Hiseq 2000/2500 instrument. Raw sequence data were processed using the manufacturer's software and quality-filtering algorithms. After demultiplexing, quality control and adapter trimming, 205,282,144 paired-end reads, 17,428,002 mate-paired (3–5 kb) reads and approximately 890 × coverage reads were obtained (we used a 10-Mb genome size to calculate the genome coverage of the sequencing output). The genome assembly of these reads was performed with SOAPdenovo2^24^ using filtered reads (Q > 20) and resulted in 9.7 Mb of sequence data in 9 contigs, in which the 36-kb contiguous contig represented the complete sequence of the plasmid pZPM234 (Fig. 1B). The order and orientations of the other 8 contigs were determined using the mate-pair reads, and the internal gaps were filled by additional genomic sequencing data from mutants using GapFiller^25^ and were then manually checked. Open reading frames (ORFs) were predicted with Glimmer3.02^26^ and Prodigal.v2^27^. The programs were trained with ~400,000 ORFs from the completely sequenced *Streptomycetes* genes that were available in public databases. We also used FramPlot29, BLAST30 (National Center for Biotechnology Information BLAST package; ftp://ftp.ncbi.nih.gov/), and HmmPfam31 to confirm the protein-coding genes predicted by Glimmer and Prodigal. CDS annotation was based on the BLASTP program with the NR, CDD and COG databases. Blast2GO was also used to identify GO annotations of the proteins^28^. *S. albulus* ZPM was annotated with GO terms, enrichment analysis was subsequently performed for specific and core genes, and Fisher's Exact test was performed with a P-value cutoff of 0.01. Phosphopantetheine binding (GO: 0031177) and DNA integration (GO: 0015074) were significantly enriched in the specific genes, with corrected P-values by False Discovery Rate (FDR) control of 2.0e-17 and 2.4e-4, respectively. tRNA and rRNA were predicted by tRNA-Scan^29^ and rRNAmmer^30^, respectively. Pair-wise alignments between *S. albulus* ZPM genome and other *Streptomycetes* genomes were performed using the Nucmer or Promer programs of the MUMmer package. The metabolic pathway was constructed based on the Kyoto Encyclopedia of Genes and Genomes (KEGG), and COG functional classification for all of the nonsynonymous variants was performed.

### Analytical method for determining the ε-PL concentration

To measure the ε-PL concentration by the method of Itzhaki^14^, a 0.1 mM phosphate buffer (prepared by adjusting the pH of a 15.6 g/l NaH_2_PO_4_·2H_2_O solution to 6.6 with a 35.8 g/l Na_2_HPO_3_·12 H_2_O solution) and a 0.1 mM methyl orange solution (Sigma) were prepared. Then, 1.9 ml of phosphate buffer and 2.0 ml of methyl orange solution were added to 0.1 ml of the supernatant. The mixtures were then vigorously reacted on a reciprocal shaker at 30°C and then centrifuged. The optical density of the resulting supernatant was measured at 465 nm, and the ε-PL concentration was calculated from the calibration curve.

### Library pooling and high-throughput sequencing

One hundred eighty mutant strains were obtained using multiple mutagenesis strategies. Of these strains, 30 that produced the lowest yield of ε-PL and 30 that produced the highest yield of ε-PL were chosen to be the low (L) and high (H) groups, respectively. For each strain, the gDNA was extracted with the UltraClean Microbial DNA Isolation Kit and checked by gel electrophoresis, and the DNA concentration was determined using a NanoDrop 2500. For each group, equal amounts of gDNA from each of the 30 strains were pooled, resulting in two DNA mixtures. Then, the DNA mixtures were fragmented using the Bioruptor standard module, and paired-end DNA sequencing libraries with an insert size of ~300 bp were constructed using the NextFlex DNA-seq Library Kit as described in the manufacturer's manuals. DNA sequencing was performed on an Illumina HiSeq 2000 sequencer according to standard protocols. The Illumina base-calling pipeline was used to process the raw fluorescent images and to call sequences. Raw reads were cleaned using in-house scripts, and low-quality reads from the paired-end sequencing were discarded.

### SNP calling and functional annotation

After de-duplication and quality filtering, 19,762,419 and 16,918,601 high-quality read pairs (2 × 100) were obtained for group-L and group-H, respectively. These short reads were aligned to the *S. albulus* ZPM reference genome sequence using Bowtie2^31^, and SNP-calling was performed. Based on the alignment, 92.5% and 95.1% of the reads were aligned exactly 1 time, and the overall alignment rates were 98.3% for group-L and 98.8% for group-H, respectively. Pileups were generated using samtools^32^ and were directly piped to VarScan2^33^ to perform pooled-library SNP calling. Low-quality read alignments (MAPQ < 10) and/or bases (BaseQ < 20) were discarded from the pileup. The minimal coverage (--min-coverage), minimal variant frequency (--min-var-freq) and strand filter (--strand-filter) of VARSCAN2 were set to be 50, 0.03, and true, respectively. Functional annotation of the SNPs, including determining whether the SNPs caused protein coding changes and identifying the affected amino acids, was performed using ANNOVAR^34^.

### Cloning, expression and purification of the recombinant proteins

*S. albulus* ZPM genes were amplified from gDNA using PCR primers. The corresponding genes were cloned into the pET28a over-expression vector to produce recombinant vectors, and protein expression and purification were performed according to the previously described procedure^35^. The elutions were dialyzed overnight and stored at −80°C, and the protein concentrations were measured using Coomassie Brilliant Blue assays^36^.

### Electrophoretic mobility shift assay (EMSA)

The binding of transcriptional regulators to the putative promoter (500 bp) of the *pls* gene was examined by EMSAs as described^35^. The HrdD coding sequence was amplified from the gDNA of *S. albulus ZPM* using specific primers and was cloned into pET28(a) expression vector. The DNA substrates of the putative *pls* promoter (pls-p500) were obtained by PCR and used in the EMSA assays. A 500 bp DNA segment of *gfp* gene (gfp-500) coding green fluorescent protein(GFP) was used as negative control. 3 nM of the DNA fragments was incubated at 4°C for 30 min or 1 hr with various amounts of proteins in a total volume of 25 μl of EMSA buffer consisting of 50 mM Tris-HCl, pH 7.5, 10 mM MgCl_2_, 1 mM DTT, and 50 mM NaCl. Then, the mixtures were directly subjected to 5% native PAGE containing 0.5 × Tris-borate-EDTA (TBE) buffer, and electrophoresis was performed at 200 V at 4°C or in an ice bath until the bromophenol blue dye reached the bottom of the gel. The gels were then stained with ethidium bromide for ~5 minutes, and the images were acquired using a Gel Doc XR Snapper (Bio-Rad). In the competition assay, 3 nM pls-p500 DNA fragments labeled with fluorescein isothiocyanate (FITC) were incubated with 0.4 μM his-tagged HrdD protein together with 5, 10 and 20 times excessive DNA substrates of unlabeled pls-p500 or gfp-500. After 1 hr incubation and further electrophoresis, the image was acquired with Typhoon Scanner (GE healthcare).

### Accession numbers

The whole-genome sequencing projects described in this study have been deposited at GenBank under accession number CP006871.

## Author Contributions

Q.W. designed the experiments. L.W., C.G. and N.T. performed the experiments. L.W., C.G., S.H. and Q.W. analyzed the results. L.W., C.G. and Q.W. wrote the manuscript.

## Supplementary Material

Supplementary Information Supplementary file

## Figures and Tables

**Table 1:** Gene clusters for secondary metabolites in *S. albulus* ZPM

Clusters	Type	From	To
1	Nrps-t4pks	17026	87092
2	Unknown	67864	109753
3	Butyrolactone	330907	341776
4	T1pks	364054	412441
5	Unknown	433815	474768
6	Nrps-t1pks-oligosaccharide	526610	600593
7	Unknown	596797	640057
8	Unknown	883066	926491
9	Lantipeptide	954336	978788
10	T1pks	1103165	1150604
11	Nrps	1137227	1196791
12	Nrps	1404109	1468194
13	Nrps-t1pkstransatpks	1476172	1525894
14	T1pks-t4pks	1670949	1783018
15	Bacteriocin	1838897	1849184
16	T2pks	1896764	1939279
17	Siderophore	2770965	2782767
18	Siderophore	2865668	2880351
19	Ectoine	3020511	3031683
20	Terpene	5358015	5380444
21	Unknown	6045012	6089448
22	T4pks-nrps-transatpks	6538252	6638419
23	T3pks	7015396	7056448
24	Nrps-butyrolactone	7056728	7124818
25	Bacteriocin	7169080	7179982
26	Oligosaccharide	7198268	7222581
27	Lantipeptide	7528854	7553415
28	T2pks-oligosaccharide	7788124	7853180
29	Nrps-nucleoside	7848120	7894680
30	Terpene	8001726	8028454
31	pls biosynthesis gene cluster(PBC)	8283445	8326182
32	Butyrolactone	8585464	8596462
33	Nrps	8706076	8762919
34	Lantipeptide	8781455	8823243
35	Butyrolactone	8870590	8881612
36	Unknown	9116811	9160601
37	T1pks-lantipeptide	9192594	9347388
38	Terpene	9329502	9379196
39	T1pks	9444073	9491961
40	Unknown	9485986	9526705
41	Lantipeptide	9545426	9574401
42	T1pks	9614130	9661965
43	Unknown	9674825	9716714
44	T4pks-Nrps	9697486	9767552

**Table 2:** Classification of low- and high-specific variants

#lib	low-specific	high-specific
**non-coding region**	**89**	**57**
intergenic	7	5
upstream	23	11
downstream	28	19
upstream; downstream	31	22
**coding region**	**119**	**106**
synonymous SNV	25	22
nonsynonymous SNV	86	81
frameshift deletion	3	1
frameshift insertion	2	2
nonframeshift deletion	1	0
nonframeshift insertion	1	0
stopgain SNV	1	0
stoploss SNV	0	0
**Total**	**208**	**163**

## References

[b1] Berdy J. Thoughts and facts about antibiotics: where we are now and where we are heading. J Antibiot (Tokyo) 65, 385–395 (2012). 22511224 10.1038/ja.2012.27

[b2] Liu G., Chater K. F., Chandra G., Niu G. & Tan H. Molecular regulation of antibiotic biosynthesis in streptomyces. Microbiol Mol Biol Rev 77, 112–143 (2013). 23471619 10.1128/MMBR.00054-12 PMC3591988

[b3] Yoshida T. & Nagasawa T. epsilon-Poly-L-lysine: microbial production, biodegradation and application potential. Appl Microbiol Biotechnol 62, 21–26 (2003). 12728342 10.1007/s00253-003-1312-9

[b4] Shima S. & Sakai H. Polylysine Produced by Streptomyces. Agric Biol Chem 41, 1807–1809 (1977).

[b5] Shima S., Matsuoka H., Iwamoto T. & Sakai H. Antimicrobial action of epsilon-poly-L-lysine. J Antibiot (Tokyo) 37, 1449–1455 (1984). 6392269 10.7164/antibiotics.37.1449

[b6] Hamano Y. *et al.* Biological function of the pld gene product that degrades epsilon-poly-L-lysine in Streptomyces albulus. Appl Microbiol Biotechnol 72, 173–181 (2006). 16568315 10.1007/s00253-006-0396-4

[b7] Hamano Y. Occurrence, biosynthesis, biodegradation, and industrial and medical applications of a naturally occurring epsilon-poly-L-lysine. Biosci Biotechnol Biochem 75, 1226–1233 (2011). 21737945 10.1271/bbb.110201

[b8] Li S. *et al.* Isolation and characterization of a novel epsilon-poly-L-lysine producing strain: Streptomyces griseofuscus. J Ind Microbiol Biotechnol 38, 557–563 (2011). 20711627 10.1007/s10295-010-0803-9

[b9] Chen X. *et al.* Optimization of medium for enhancement of epsilon-poly-L-lysine production by Streptomyces sp. M-Z18 with glycerol as carbon source. Bioresour Technol 102, 1727–1732 (2011). 20846854 10.1016/j.biortech.2010.08.071

[b10] Liu S. R., Wu Q. P., Zhang J. M. & Mo S. P. Production of epsilon-poly-L-lysine by Streptomyces sp using resin-based, in situ product removal. Biotechnol Lett 33, 1581–1585 (2011). 21720848 10.1007/s10529-011-0616-6

[b11] Ouyang J. *et al.* Production of epsilon-poly-L-lysine by newly isolated Kitasatospora sp. PL6-3. Biotechnol J 1, 1459–1463 (2006). 17161019 10.1002/biot.200600079

[b12] Yamanaka K., Maruyama C., Takagi H. & Hamano Y. epsilon-Poly-L-lysine dispersity is controlled by a highly unusual nonribosomal peptide synthetase. Nat Chem Biol 4, 766–772 (2008). 18997795 10.1038/nchembio.125

[b13] Yamanaka K. *et al.* Mechanism of epsilon-Poly-L-Lysine Production and Accumulation Revealed by Identification and Analysis of an epsilon-Poly-L-Lysine-Degrading Enzyme. Appl Environ Microbiol 76, 5669–5675 (2010). 20601519 10.1128/AEM.00853-10 PMC2935060

[b14] Kahar P., Iwata T., Hiraki J., Park E. Y. & Okabe M. Enhancement of epsilon-polylysine production by Streptomyces albulus strain 410 using pH control. J Biosci Bioeng 91, 190–194 (2001). 16232973 10.1263/jbb.91.190

[b15] Nishikawa M. & Ogawa K. Distribution of microbes producing antimicrobial epsilon-poly-L-lysine polymers in soil microflora determined by a novel method. Appl Environ Microbiol 68, 3575–3581 (2002). 12089045 10.1128/AEM.68.7.3575-3581.2002 PMC126757

[b16] Ohnishi Y. *et al.* Genome sequence of the streptomycin-producing microorganism Streptomyces griseus IFO 13350. J Bacteriol 190, 4050–4060 (2008). 18375553 10.1128/JB.00204-08 PMC2395044

[b17] Bentley S. D. *et al.* Complete genome sequence of the model actinomycete Streptomyces coelicolor A3(2). Nature 417, 141–147 (2002). 12000953 10.1038/417141a

[b18] Ikeda H. *et al.* Complete genome sequence and comparative analysis of the industrial microorganism Streptomyces avermitilis. Nat Biotechnol 21, 526–531 (2003). 12692562 10.1038/nbt820

[b19] Kim Y. J. *et al.* Acidic pH shock induces the expressions of a wide range of stress-response genes. BMC Genomics 9, 604 (2008). 19087294 10.1186/1471-2164-9-604 PMC2631018

[b20] Dodd A., Swanevelder D., Featherston J. & Rumbold K. Draft Genome Sequence of Streptomyces albulus Strain CCRC 11814, an Epsilon-Poly-L-Lysine-Producing Actinomycete. Genome Announc 1, e00696-13 (2013). 24009124 10.1128/genomeA.00696-13 PMC3764419

[b21] Xu Z. *et al.* Genome Sequence of Streptomyces albulus PD-1, a Productive Strain for Epsilon-Poly-L-Lysine and Poly-L-Diaminopropionic Acid. Genome Announc 2, e00297-14 (2014). 24744334 10.1128/genomeA.00297-14 PMC3990750

[b22] Alper H., Moxley J., Nevoigt E., Fink G. R. & Stephanopoulos G. Engineering yeast transcription machinery for improved ethanol tolerance and production. Science 314, 1565–1568 (2006). 17158319 10.1126/science.1131969

[b23] Steensels J. *et al.* Improving industrial yeast strains: exploiting natural and artificial diversity. FEMS Microbiol Rev 38, 947–995 (2014). 24724938 10.1111/1574-6976.12073 PMC4293462

[b24] Luo R. *et al.* SOAPdenovo2: an empirically improved memory-efficient short-read de novo assembler. Gigascience 1, 18 (2012). 23587118 10.1186/2047-217X-1-18 PMC3626529

[b25] Boetzer M. & Pirovano W. Toward almost closed genomes with GapFiller. Genome Biol 13, R56 (2012). 22731987 10.1186/gb-2012-13-6-r56 PMC3446322

[b26] Delcher A. L., Bratke K. A., Powers E. C. & Salzberg S. L. Identifying bacterial genes and endosymbiont DNA with Glimmer. Bioinformatics 23, 673–679 (2007). 17237039 10.1093/bioinformatics/btm009 PMC2387122

[b27] Hyatt D. *et al.* Prodigal: prokaryotic gene recognition and translation initiation site identification. BMC Bioinformatics 11, 119 (2010). 20211023 10.1186/1471-2105-11-119 PMC2848648

[b28] Conesa A. *et al.* Blast2GO: a universal tool for annotation, visualization and analysis in functional genomics research. Bioinformatics 21, 3674–3676 (2005). 16081474 10.1093/bioinformatics/bti610

[b29] Lowe T. M. & Eddy S. R. tRNAscan-SE: A program for improved detection of transfer RNA genes in genomic sequence. Nucl Acids Res 25, 955–964 (1997). 9023104 10.1093/nar/25.5.955 PMC146525

[b30] Lagesen K. *et al.* RNAmmer: consistent and rapid annotation of ribosomal RNA genes. Nucl Acids Res 35, 3100–3108 (2007). 17452365 10.1093/nar/gkm160 PMC1888812

[b31] Langmead B., Trapnell C., Pop M. & Salzberg S. L. Ultrafast and memory-efficient alignment of short DNA sequences to the human genome. Genome Biol 10, R25 (2009). 19261174 10.1186/gb-2009-10-3-r25 PMC2690996

[b32] Li H. *et al.* The Sequence Alignment/Map format and SAMtools. Bioinformatics 25, 2078–2079 (2009). 19505943 10.1093/bioinformatics/btp352 PMC2723002

[b33] Koboldt D. C. *et al.* VarScan 2: Somatic mutation and copy number alteration discovery in cancer by exome sequencing. Genome Res 22, 568–576 (2012). 22300766 10.1101/gr.129684.111 PMC3290792

[b34] Wang K., Li M. Y. & Hakonarson H. ANNOVAR: functional annotation of genetic variants from high-throughput sequencing data. Nucl Acids Res 38, e164 (2010). 20601685 10.1093/nar/gkq603 PMC2938201

[b35] Gao C. H., Yang M. & He Z. G. Characterization of a Novel ArsR-Like Regulator Encoded by Rv2034 in Mycobacterium tuberculosis. Plos One 7, e36255 (2012). 22558408 10.1371/journal.pone.0036255 PMC3338718

[b36] Bradford M. M. A rapid and sensitive method for the quantitation of microgram quantities of protein utilizing the principle of protein-dye binding. Anal Biochem 72, 248–254 (1976). 942051 10.1016/0003-2697(76)90527-3

