# Supplementary material for: Identification of genetic variations associated with epsilon-poly-lysine biosynthesis in Streptomyces albulus ZPM by genome sequencing
Source: Sci Rep. 2015 Mar 17;5:9201. doi: 10.1038/srep09201 (PMC4361855; doi:10.1038/srep09201)
Supplement: Supplementary Information — Supplementary file [file srep09201-s1.pdf]

Supplementary information

Identification of genetic variations associated with epsilon-poly-lysine biosynthesis in *Streptomyces albulus ZPM* by genome sequencing

Lin Wang[1,#], Chunhui Gao[1, #], Nan Tang[1], Songnian Hu[3], Qingfa Wu[1,2,*]

1. School of Life Sciences and Medical Center, University of Science & Technology of China, Hefei, Anhui 230027, China
2. CAS Key Laboratory of Innate Immunity and Chronic Disease, School of Life Sciences and Medical Center, University of Science & Technology of China, Hefei, Anhui 230027, China
3. CAS Key Laboratory of Genome Sciences & Information, Beijing institute of Genomics, CAS, 100101

#. These authors contributed equally to this work.
* Corresponding authors. wuqf@ustc.edu.cn

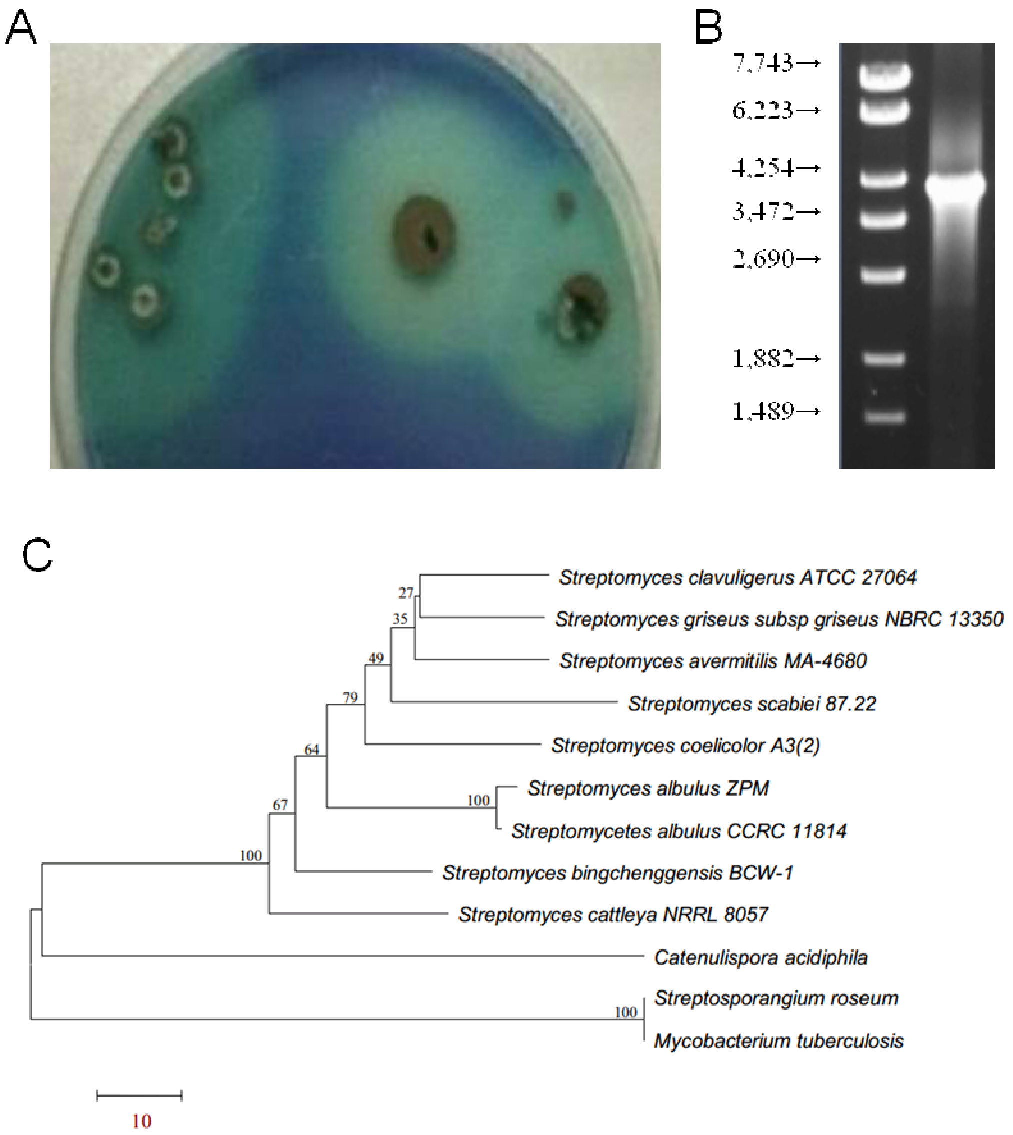

Figure S1. ε-PL-producing strain isolation based on the methylene blue method, molecular identification of the ε-PL synthesis enzyme gene and phylogenetic analysis of the 16S rRNAs of *Streptomyces* species.

(A) Isolation of the ε-PL strain on agar plates embedded with the charged dye methylene blue. (B) PCR of the ε-PL synthesis using specific primer pairs. (C) Phylogenetic analysis of the 16S rRNAs of *Streptomyces* species. The name *S. albulus* ZPM was given because the bacteria has the closest relationship with *S. albulus* CCRC 11814. The bootstrap values are shown on the branch.

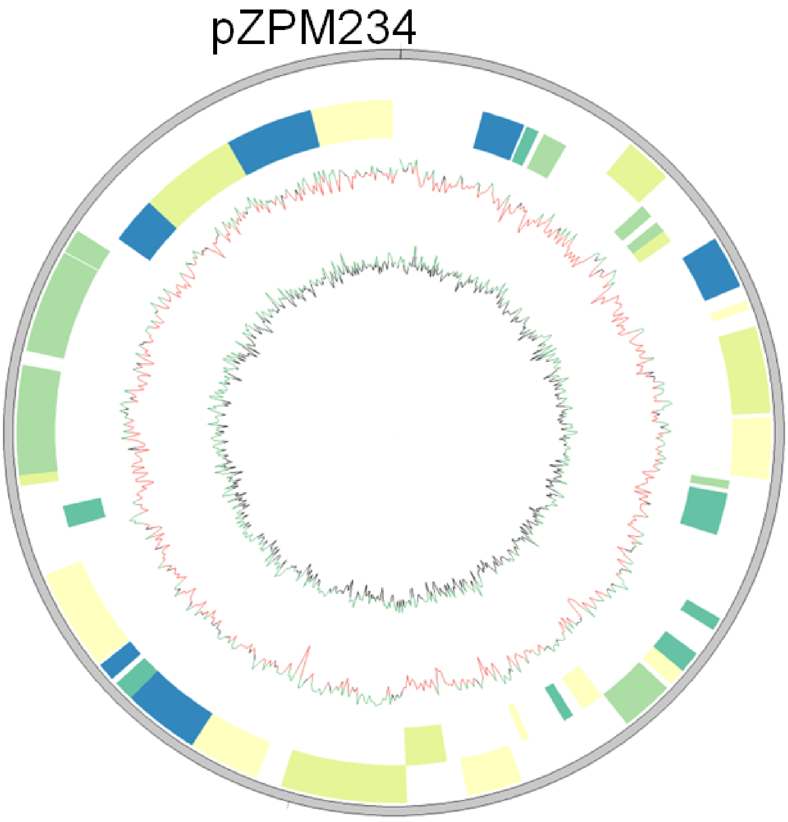

Figure S2. Circular representation of pZPM234. Circles 1 and 2, predicted protein-coding sequences (forward and reverse strands); Circle 3, GC content; Circle 4, GC bias.

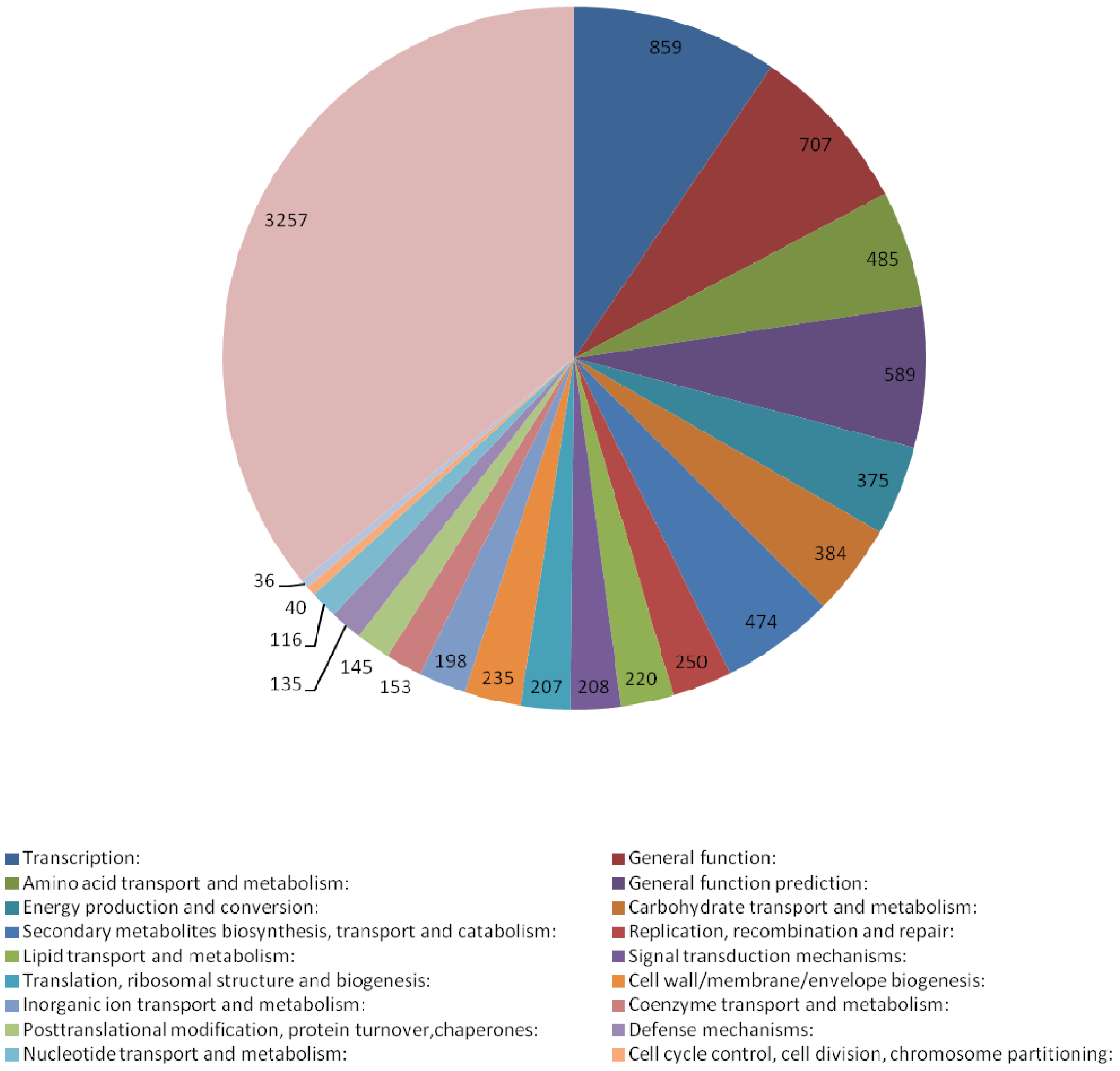

Figure S3. **COG classification of 9073 genes identified in *S. albulus* ZPM.** In total, 5816 of 9073 protein-coding genes (64.1%) have been classified into at least one COG group with known or putative function.

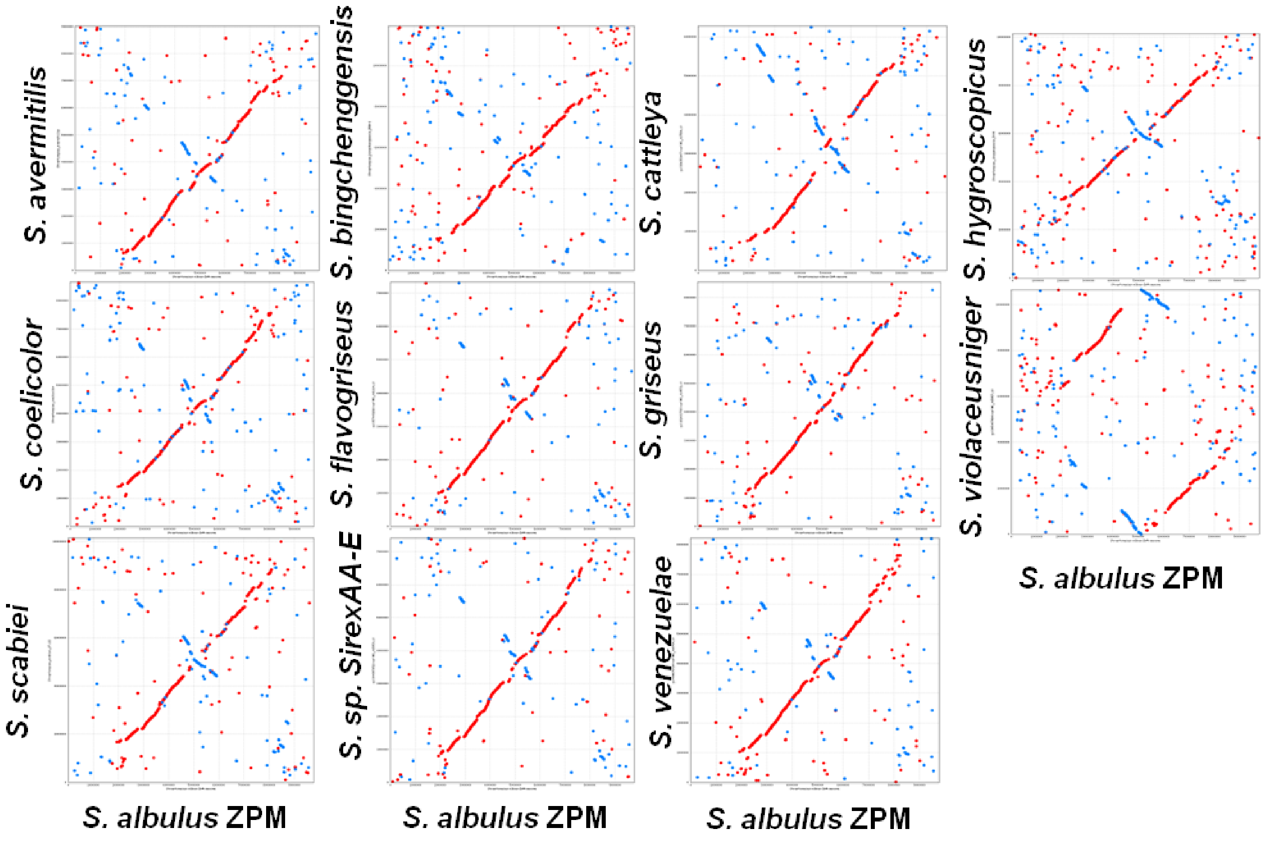

Figure S4. Dot plots of whole-genome comparisons of *S. albulus* ZPM with other *Streptomyces* species. The whole-genome comparisons between *S. albulus* ZPM and *S. coelicolor, S. griseus, S. avermitilis, S. bingchenggensis, S. flavogriseus, S. sp. SirexAA-E, S. violaceusniger, S. cattleya, S. hygroscopicus, S. scabiei, S. clavuligerus* and *S. venezuelae* were performed and visualized using the Mummer program.

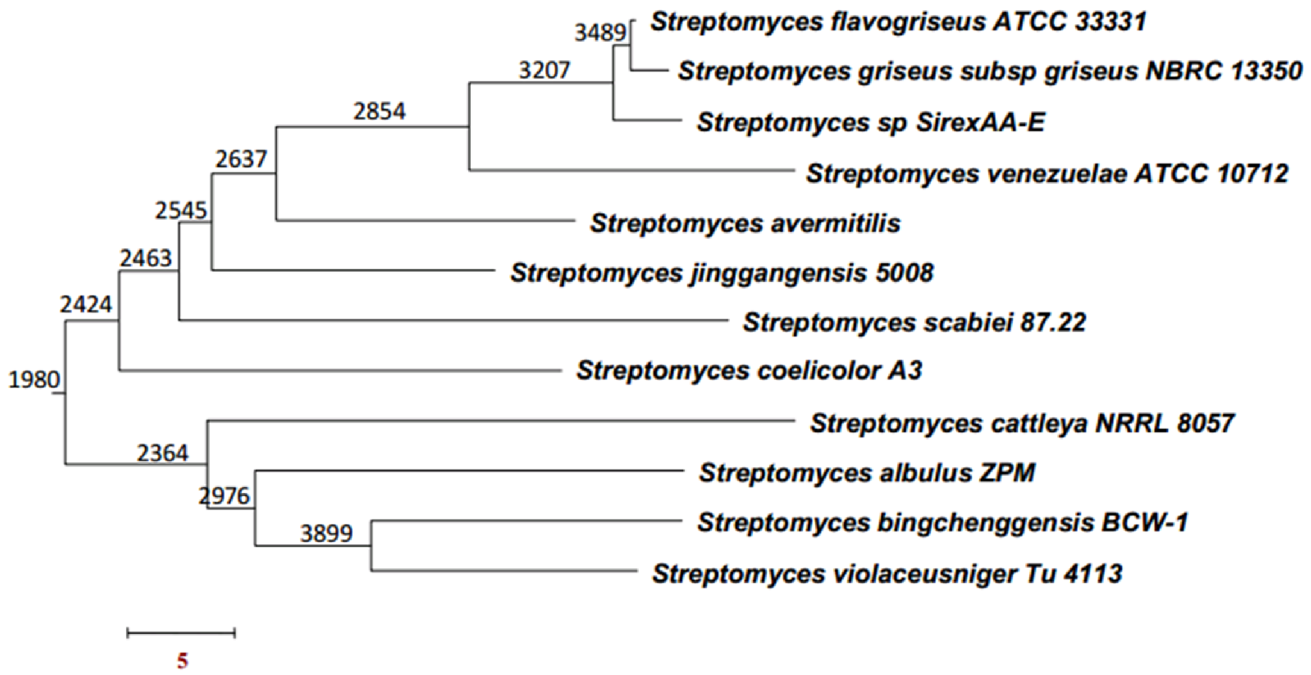

Figure S5. Lineage-specific gene analysis using a phylogenetic tree. The phylogenetic tree was constructed using the neighbor-joining method and was based on 16S rRNA sequences. The number of lineage-specific genes of each node is indicated on the evolutionary branches.

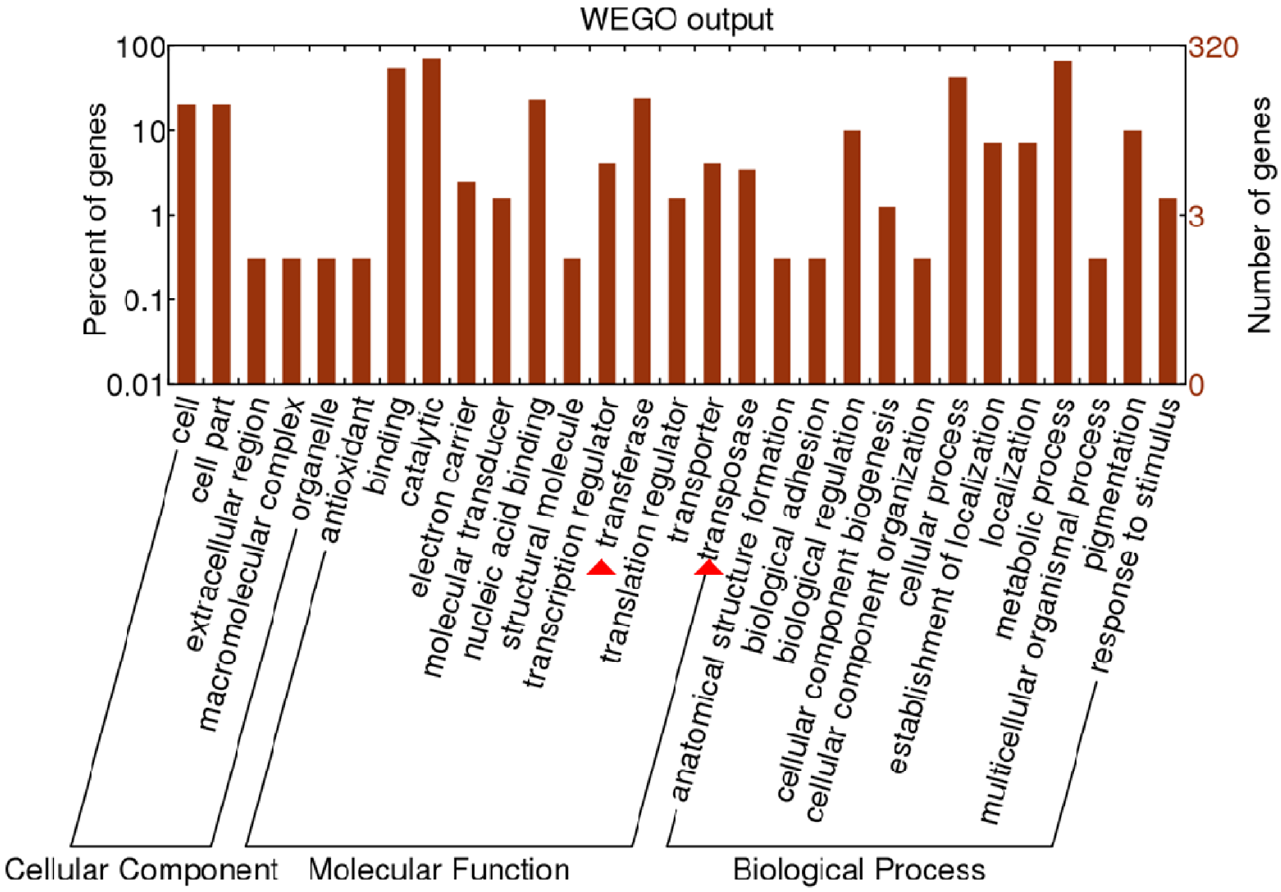

Figure S6. GO enrichment analysis of the specific genes in *S. albulus* ZPM. The classes of phosphopantetheinyl transferase (GO: 0016740) and DNA transposase (GO: 0004803) in the Molecular Function domain are enriched for the specific genes of *S. albulus* ZPM.

Table S1 General features of the chromosome of *S. albulus* ZPM

Component of the chromosome	Property
Total size	9,784,577 bp
Terminal inverted repeat	139,385 bp
G+C content	72.2%
RNA	76
rRNA (16S-23S-5S)	6
tRNA	70 (48 species)
ORFs	9073
Classified in at least one COG group	5816 (64.1%)
Having specific CDD hit(s)	6811 (75.0%)
Coding density	87.5%
Average CDS length	944

Table S2 Pan-genome analysis between *S. albulus* ZPM and other *Streptomyces* species

Organism	CDS	Core CDS	Var CDS	Strain-specific CDS	Core CDS (%)	Var CDS (%)	Strain-specific CDS (%)
S. albulus ZPM	9073	3985	2916	2172	43.92	32.14	23.94
**S. avermitilis* MA 4680	7582	3756	2826	1000	49.54	37.27	13.19
**S. cattleya* NRRL 8057	5822	3105	1467	1250	53.33	25.20	21.47
**S. coelicolor* A3 (2)	7825	3878	3077	870	49.56	39.32	11.12
**S. venezuelae* ATCC 10712	7453	3588	2718	1147	48.14	36.47	15.39
**S. scabiei* 88.02	8746	3889	3386	1471	44.47	38.71	16.82
**S. violaceusniger* Tu 4113	8482	4176	3357	949	49.23	39.58	11.19
**S. bingchenggensis* BCW-1	10023	4555	3628	1840	45.45	36.20	18.36
**S. sp* SirexAA-E	6357	3535	2405	417	55.61	37.83	06.56
**S. flavogriseus* ATCC 33331	6298	3480	2438	380	55.26	38.71	06.03
**S. hygroscopicus* 5008	8849	4014	3306	1529	45.36	37.36	17.28
**S. griseus*	7138	3626	2696	816	50.80	37.77	11.43

*Data were obtained from GenBank: *S. avermitilis* MA-4680 (NC_003155); *S. cattleya* NRRL 8057 (NC_017586); *S. coelicolor* A3 (2) (NC_003888); *S. venezuelae* ATCC 10712 (NC_018750); *S. scabiei* 88.22 (NC_013929); *S. violaceusniger* Tu 4113 (NC_015957); *S. bingchenggensis* BCW-1 (NC_016582); *S. sp* SirexAA-E (NC_015953); *S. flavogriseus* ATCC 33331 (NC_016114); *S. hygroscopicus* 5008 (NC_017765); and *S. griseus* 13350 (NC_010572).

Table S3. The yield of each strain in group-L and group-H

Group-L		Group-H	
No.	Yield (g/L)	No.	Yield (g/L)
1	0.2353	1	1.05916
2	0.2331	2	1.02336
3	0.2197	3	1.0167
4	0.213	4	1.007
5	0.2108	5	0.9786
6	0.1548	6	0.909
7	0.1257	7	0.8913
8	0.1212	8	0.8845
9	0.1167	9	0.8689
10	0.0943	10	0.8644
11	0.0781	11	0.8406
12	0.0541	12	0.8384
13	0.0451	13	0.8362
14	0.0339	14	0.8339
15	0.022	15	0.8317
16	0.0205	16	0.8205
17	0.016	17	0.8183
18	0.0131	18	0.8116
19	0.0086	19	0.8115
20	0.0071	20	0.8106
21	0.0019	21	0.8071
22	0	22	0.807
23	0	23	0.8062
24	0	24	0.8049
25	0	25	0.8039
26	0	26	0.8026
27	0	27	0.8026
28	0	28	0.8017
29	0	29	0.8004
30	0	30	0.7985

Table S4. All genetic variations identified in both group-H and group-L

No.	Chromosomal position (nt)	Ref.	Genetic variations	Frequencies of genetic variations		genetic variations of coding regions			genetic variations of non-coding regions	
				group-L	group-H	Affected genes	Type	Amino acid change	Nearest genes	Positions to genes
**1**	218244	T	C	100%	100%	SAZ_0187	Ns	E62G		
**2**	237916	T	G	3.27%	-				SAZ_0210,SAZ_0209	Up, Dn
**3**	270391	A	C	-	3.50%	SAZ_0249	Ns	Y72S		
**4**	279849	-	C	13.92%	17.12%				SAZ_0258	Dn
**5**	343098	A	C	3.04%	-	SAZ_0323	Ns	H259P		
**6**	400188	A	C	3.27%	-	SAZ_0379	Ns	H289P		
**7**	440541	A	C	3.16%	-				SAZ_0427,SAZ_0428	Up
**8**	455109	T	G	-	4.27%				SAZ_0440	Dn
**9**	505143	T	G	3.86%	-	SAZ_0496	Ns	T258P		
**10**	584310	T	G	3.29%	-	SAZ_0569	Ns	H275P		
**11**	635013	T	G	3.23%	-	SAZ_0612	Ns	H214P		
**12**	643291	G	C	6.09%	-				SAZ_0618,SAZ_0619	Up
**13**	717229	C	T	5.23%	-	SAZ_0692	Ns	R316H		
**14**	725634	C	G	8.65%	-	SAZ_0698	S	G371G		
**15**	725653	C	G	7.69%	-				SAZ_0698,SAZ_0699	Dn
**16**	750584	A	C	3.76%	-	SAZ_0722	S	A308A		
**17**	794578	G	C	4.89%	-				SAZ_0770,SAZ_0771	Dn
**18**	829350	G	A	99.12%	100%	SAZ_0807	S	L94L		
**19**	829393	C	G	98.96%	100%	SAZ_0807	Ns	R109G		
**20**	865573	T	C	98.89%	100%	SAZ_0848	Ns	K15E		
**21**	874584	A	C	-	3.76%				SAZ_0858	Dn
**22**	887570	A	C	-	3.50%	SAZ_0870	Ns	S15R		
**23**	890573	A	C	-	3.76%	SAZ_0877	Ns	Q272P		
**24**	900483	A	C	3.36%	7.01%				SAZ_0889,SAZ_0890,SAZ_0888	Up, Dn
**25**	982662	A	C	4.76%	-	SAZ_0972	Ns	T13P		
**26**	1019712	C	T	-	3.51%	SAZ_1010	Ns	G590D		
**27**	1023457	T	G	4.57%	-	SAZ_1011	Ns	V529G		
**28**	1023732	G	A	-	7.08%	SAZ_1011	Ns	V621I		

**29**	1026677	A	C	7.19%	7.53%	SAZ_1014	Ns	H77P		
**30**	1029236	T	G	3.02%	-	SAZ_1016	Ns	T307P		
**31**	1053304	T	G	3.38%	-	SAZ_1045	S	A63A		
**32**	1073978	A	C	-	3.11%	SAZ_1062	Ns	T527P		
**33**	1102650	C	G	6.11%	-				SAZ_1092,SAZ_1093	IR
**34**	1102657	C	G	9.62%	-				SAZ_1092,SAZ_1093	IR
**35**	1104927	G	C	8.33%	10.59%				SAZ_1096,SAZ_1095	Up, Dn
**36**	1104936	G	C	13.54%	13.64%				SAZ_1096,SAZ_1095	Up, Dn
**37**	1127129	A	C	-	3%	SAZ_1118	Ns	V1147G		
**38**	1152312	C	A	34.63%	29.25%	SAZ_1139	S	V141V		
**39**	1152351	C	G	32.02%	28.22%	SAZ_1139	S	A128A		
**40**	1152661	C	G	5.26%	6.39%	SAZ_1139	Ns	S25T		
**41**	1152666	G	A	3.35%	3.33%	SAZ_1139	S	D23D		
**42**	1155091	C	G	-	13.46%	SAZ_1141	S	P212P		
**43**	1195075	T	G	-	3.39%	SAZ_1165	Ns	T180P		
**44**	1223137	T	G	3.36%	-	SAZ_1195	Ns	N8T		
**45**	1257970	A	C	-	4.61%	SAZ_1227	Ns	T57P		
**46**	1258765	T	G	-	3.10%	SAZ_1229	Ns	N357H		
**47**	1321149	A	C	-	3.18%	SAZ_1284	Ns	V116G		
**48**	1352616	C	G	4.21%	-	SAZ_1312	S	A46A		
**49**	1428347	G	C	100%	100%	SAZ_1375	Ns	A1197G		
**50**	1428352	G	C	100%	100%	SAZ_1375	Ns	D1195E		
**51**	1429342	G	A	45%	38%	SAZ_1375	S	S865S		
**52**	1431395	T	C	4.97%	6.33%	SAZ_1375	Ns	Q181R		
**53**	1431598	G	A	13.87%	14.62%	SAZ_1375	S	D113D		
**54**	1431612	G	A	12.65%	12.65%	SAZ_1375	S	L109L		
**55**	1431639	G	A	8.93%	10.09%	SAZ_1375	S	L100L		
**56**	1431641	G	A	8.06%	8.72%	SAZ_1375	Ns	A99V		
**57**	1431654	G	C	3.66%	6.94%	SAZ_1375	Ns	P95A		
**58**	1431657	G	-	3.73%	3.93%	SAZ_1375	FS	R94fs		
**59**	1446132	T	G	-	3.88%	SAZ_1389	Ns	F331C		
**60**	1465995	-	G	7.04%	4.35%	SAZ_1406	FS	M5fs		
**61**	1479016	T	G	3.57%	-	SAZ_1419	Ns	T385P		
**62**	1540684	T	G	-	5.07%	SAZ_1473	Ns	T117P		
**63**	1602208	A	C	-	3.06%	SAZ_1530	Ns	T357P		

**64**	1614211	C	T	42.64%	41.84%	SAZ_1542	Ns	E243K		
**65**	1621069	C	G	100%	100%				SAZ_1549,SAZ_1550	Up
**66**	1621072	C	G	100%	98.73%				SAZ_1549,SAZ_1550	Up
**67**	1632665	C	T	5.08%	-	SAZ_1564	Ns	P103S		
**68**	1634603	T	G	16.42%	-				SAZ_1565,SAZ_1566	Up, Dn
**69**	1683141	G	C	7.64%	-				SAZ_1611	Up
**70**	1683155	G	C	6.21%	-				SAZ_1611	Up
**71**	1683161	G	C	14.89%	7.78%				SAZ_1611	Up
**72**	1685295	A	C	3.61%	-	SAZ_1614	Ns	T103P		
**73**	1703572	G	A	-	6.86%	SAZ_1623	S	A1612A		
**74**	1722049	-	GG	3.70%	4.92%				SAZ_1625,SAZ_1626	Dn
**75**	1764863	-	A	25.78%	20.20%	SAZ_1632	FS	P811fs		
**76**	1775973	C	G	3.62%	10.86%	SAZ_1636	S	G424G		
**77**	1787509	A	C	-	3.56%	SAZ_1645	Ns	V37G		
**78**	1813141	C	G	13.56%	-	SAZ_1669	Ns	A164P		
**79**	1813154	C	G	33.33%	-	SAZ_1669	S	P159P		
**80**	1826689	G	C	3.70%	-				SAZ_1683,SAZ_1682	Up, Dn
**81**	1850944	G	A	11.65%	22.09%	SAZ_1705	S	T434T		
**82**	1864425	T	G	-	6.08%	SAZ_1718	Ns	T134P		
**83**	1864816	C	A	42.67%	38.96%	SAZ_1718	S	P3P		
**84**	1908546	A	C	-	3.69%	SAZ_1760	Ns	V297G		
**85**	1974343	T	G	3.48%	-				SAZ_1829,SAZ_1828	Up, Dn
**86**	1978485	-	GA	-	12.50%	SAZ_1831	FS	T1140fs		
**87**	1978496	G	A	45.16%	59.62%	SAZ_1831	Ns	T1136I		
**88**	1978508	C	-	13.04%	19.61%	SAZ_1831	FS	G1132fs		
**89**	2029888	T	G	4.69%	-	SAZ_1881	Ns	H207P		
**90**	2057581	A	-	95.97%	92.72%				SAZ_1908	Up
**91**	2057601	T	C	100%	99.21%				SAZ_1908	Up
**92**	2057730	TGTTCG	-	77.85%	77.57%				SAZ_1908	Up
**93**	2073702	C	G	4.86%	-				SAZ_1918,SAZ_1917	Up, Dn
**94**	2079602	A	G	100%	100%	SAZ_1923	Ns	Q221R		
**95**	2079610	A	G	98.65%	100%	SAZ_1923	Ns	T224A		
**96**	2079620	G	T	98.28%	94%	SAZ_1923	Ns	G227V		
**97**	2135830	T	G	-	3.59%				SAZ_1973,SAZ_1974	Up, Dn
**98**	2146091	A	C	-	3.10%	SAZ_1981	Ns	V85G		

**99**	2188229	C	G	3.18%	-	SAZ_2020	S	P255P		
**100**	2189776	C	G	3.91%	-	SAZ_2021	Ns	A181G		
**101**	2199062	T	G	5.62%	7.38%	SAZ_2030	Ns	T483P		
**102**	2206251	C	T	31.62%	15.71%	SAZ_2036	Ns	R232Q		
**103**	2253313	T	G	-	4.46%				SAZ_2082	Dn
**104**	2253384	C	T	5.49%	-				SAZ_2082	Dn
**105**	2253414	T	C	4.18%	5.56%				SAZ_2082	Dn
**106**	2253429	C	A	-	3.43%				SAZ_2082,SAZ_2083	Dn
**107**	2253500	A	G	-	4.62%				SAZ_2082,SAZ_2083	Dn
**108**	2271512	A	C	3.78%	-				SAZ_2100,SAZ_2102,SAZ_2101	Up, Dn
**109**	2365560	G	C	6.81%	-	SAZ_2192	Ns	A106G		
**110**	2366219	G	T	3.68%	-	SAZ_2193	Ns	T7N		
**111**	2380515	G	C	98.46%	-	SAZ_2206	S	P29P		
**112**	2415088	G	A	15.32%	24.83%				SAZ_2241	Dn
**113**	2444422	G	-	-	23.08%	SAZ_2269	FS	R71fs		
**114**	2446251	T	G	-	5.30%				SAZ_2270,SAZ_2271	Up, Dn
**115**	2456829	T	G	-	3.29%	SAZ_2281	Ns	T445P		
**116**	2527518	G	A	4.25%	-				SAZ_2346,SAZ_2347	Up
**117**	2529583	G	C	6.43%	-				SAZ_2348,SAZ_2349	Dn
**118**	2563317	A	G	9.13%	6.35%	SAZ_2382	S	G937G		
**119**	2563406	G	T	86.67%	90.70%	SAZ_2382	Ns	P908T		
**120**	2563413	T	C	-	98%	SAZ_2382	S	S905S		
**121**	2563429	C	A	97.13%	99.39%	SAZ_2382	Ns	G900V		
**122**	2563446	C	G	99.56%	99.50%	SAZ_2382	S	G894G		
**123**	2563465	G	T	98.90%	99.57%	SAZ_2382	Ns	A888E		
**124**	2563478	T	C	100%	100%	SAZ_2382	Ns	M884V		
**125**	2563487	G	T	99.31%	100%	SAZ_2382	Ns	Q881K		
**126**	2563520	G	T	98.82%	99.49%	SAZ_2382	Ns	P870T		
**127**	2564046	A	C	37.22%	35.84%	SAZ_2382	S	G694G		
**128**	2564121	A	G	22.62%	26.28%	SAZ_2382	S	L669L		
**129**	2565574	C	G	32.21%	31.99%	SAZ_2382	Ns	G185A		
**130**	2578991	T	G	-	5.67%	SAZ_2395	Ns	T542P		
**131**	2601721	T	G	3.41%	3.59%				SAZ_2416,SAZ_2417	IR
**132**	2641081	A	C	-	3%	SAZ_2443	Ns	V33G		
**133**	2643120	A	C	-	3.88%	SAZ_2448	S	G149G		

**134**	2647165	T	G	99.69%	99.63%	SAZ_2458	Ns	I27M		
**135**	2670417	G	T	100%	98.08%				SAZ_2485,SAZ_2486	Up
**136**	2690067	C	G	4.94%	7.14%	SAZ_2501	Ns	R50P		
**137**	2713580	G	C	23.61%	16.39%				SAZ_2520,SAZ_2521	Dn
**138**	2713586	-	GA	7.03%	7.69%				SAZ_2520,SAZ_2521	Dn
**139**	2713603	-	C	10%	-				SAZ_2520,SAZ_2521	Dn
**140**	2713615	G	C	34.25%	36.54%				SAZ_2520,SAZ_2521	Dn
**141**	2760201	G	C	5.03%	-				SAZ_2563,SAZ_2564	Up
**142**	2760205	G	C	6.96%	7.74%				SAZ_2563,SAZ_2564	Up
**143**	2769273	T	G	6.83%	6.32%	SAZ_2573	Ns	V149G		
**144**	2769289	G	C	4.23%	6.25%	SAZ_2573	S	P154P		
**145**	2770127	A	C	4%	-	SAZ_2574	Ns	V258G		
**146**	2794384	A	G	-	3.54%	SAZ_2595	Ns	L53P		
**147**	2797669	T	C	4.02%	-	SAZ_2597	Ns	S8P		
**148**	2809067	C	A	99.49%	100%	SAZ_2610	Ns	A390S		
**149**	2809088	G	T	99.46%	100%	SAZ_2610	Ns	Q383K		
**150**	2809094	G	T	100%	100%	SAZ_2610	Ns	R381S		
**151**	2809675	A	T	99.18%	98.65%	SAZ_2610	Ns	V187D		
**152**	2809689	G	C	100%	100%	SAZ_2610	Ns	F182L		
**153**	2809692	G	C	100%	100%	SAZ_2610	Ns	D181E		
**154**	2809695	A	G	100%	98.81%	SAZ_2610	S	D180D		
**155**	2809716	T	G	100%	97.87%	SAZ_2610	S	I173I		
**156**	2809724	A	G	97.90%	98.98%	SAZ_2610	Ns	W171R		
**157**	2809727	T	G	100%	98.94%	SAZ_2610	Ns	N170H		
**158**	2809752	G	C	100%	100%	SAZ_2610	S	A161A		
**159**	2824776	C	G	7.77%	-	SAZ_2625	Ns	A756P		
**160**	2824785	T	G	20.69%	-	SAZ_2625	Ns	T753P		
**161**	2824790	C	G	17.33%	19.67%	SAZ_2625	Ns	R751P		
**162**	2824796	T	G	6.93%	-	SAZ_2625	Ns	H749P		
**163**	2825154	C	G	4.94%	6.06%	SAZ_2625	Ns	A630P		
**164**	2852711	T	G	3.63%	-	SAZ_2647	Ns	T352P		
**165**	2871855	T	G	9.58%	10.07%	SAZ_2662	Ns	T216P		
**166**	2933742	C	A	36.68%	27.66%	SAZ_2721	Ns	V170F		
**167**	2943953	A	-	94.84%	94.13%				SAZ_2725	Up
**168**	2944102	TGTTCG	-	77.84%	80.24%				SAZ_2725	Up

**169**	2955650	C	A	3.41%	3.76%	SAZ_2735	Ns	A55S		
**170**	2964936	G	T	100%	100%	SAZ_2744	Ns	P118H		
**171**	3033409	T	G	-	6.67%	SAZ_2816	Ns	T97P		
**172**	3050361	G	C	-	3.79%				SAZ_2833,SAZ_2834	Up, Dn
**173**	3105319	A	G	8.21%	-				SAZ_2885,SAZ_2884	Up, Dn
**174**	3107253	G	-	3.58%	-				SAZ_2887,SAZ_2888	Up, Dn
**175**	3131029	G	C	6.10%	-	SAZ_2908	Ns	A42P		
**176**	3152310	G	C	9.45%	-	SAZ_2922	Ns	A7P		
**177**	3157805	T	G	-	3.30%	SAZ_2928	Ns	S89R		
**178**	3158238	A	C	98.97%	100%				SAZ_2928,SAZ_2929	Up
**179**	3158254	C	A	98.15%	100%	SAZ_2929	Ns	L4M		
**180**	3158271	C	A	100%	98.86%	SAZ_2929	S	S9S		
**181**	3158301	A	G	98.75%	100%	SAZ_2929	Ns	I19M		
**182**	3158309	C	A	100%	100%	SAZ_2929	Ns	A22D		
**183**	3158316	A	G	100%	-	SAZ_2929	S	R24R		
**184**	3158320	G	T	100%	-	SAZ_2929	Ns	V26F		
**185**	3162544	C	G	-	4.58%	SAZ_2931	S	P181P		
**186**	3164873	C	G	3.73%	4.59%	SAZ_2932	Ns	A360P		
**187**	3167920	C	G	4.40%	8.45%	SAZ_2934	Ns	A281G		
**188**	3169895	T	C	3.46%	8.24%	SAZ_2935	Ns	E702G		
**189**	3210749	C	T	5.03%	-	SAZ_2977	S	P52P		
**190**	3273560	G	C	95%	-	SAZ_3031	Ns	A99G		
**191**	3273581	G	C	95.59%	100%	SAZ_3031	Ns	P92R		
**192**	3273608	G	C	99.32%	99.43%	SAZ_3031	Ns	A83G		
**193**	3304392	C	G	5.65%	9.02%				SAZ_3061,SAZ_3062	Up
**194**	3312913	A	T	6.63%	4.17%				SAZ_3069	Dn
**195**	3334355	G	C	3.10%	3.77%				SAZ_3090,SAZ_3091	Dn
**196**	3334360	G	C	8.06%	10.78%				SAZ_3090,SAZ_3091	Dn
**197**	3379969	T	G	4.20%	4.65%	SAZ_3134	Ns	H445P		
**198**	3381337	G	A	100%	100%				SAZ_3134	Up
**199**	3386874	A	C	-	7.20%	SAZ_3141	Ns	F119C		
**200**	3387184	T	G	3.06%	8.51%	SAZ_3141	Ns	T16P		
**201**	3387193	T	G	-	4.40%	SAZ_3141	Ns	T13P		
**202**	3391862	G	C	29.41%	31.25%				SAZ_3146,SAZ_3145	Up, Dn
**203**	3391866	G	C	20.34%	16.13%				SAZ_3146,SAZ_3145	Up, Dn

**204**	3391870	T	C	-	19.70%				SAZ_3146,SAZ_3145	Up, Dn
**205**	3408950	A	C	-	4.11%	SAZ_3160	Ns	V303G		
**206**	3409834	C	G	-	8.82%	SAZ_3160	S	P8P		
**207**	3409842	C	G	11.67%	-	SAZ_3160	Ns	A6P		
**208**	3409878	C	G	-	9.41%				SAZ_3160,SAZ_3161	Up, Dn
**209**	3409885	C	G	-	9.76%				SAZ_3160,SAZ_3161	Up, Dn
**210**	3438760	A	C	-	3.07%	SAZ_3190	Ns	L345R		
**211**	3443728	G	C	5.30%	-				SAZ_3193,SAZ_3194	Up
**212**	3443734	G	C	4.05%	-				SAZ_3193,SAZ_3194	Up
**213**	3501824	GAT	-	21.62%	18.42%	SAZ_3258	nonFS	361_361del		
**214**	3507525	C	G	5.19%	-				SAZ_3263	Up
**215**	3507529	C	G	10.40%	-				SAZ_3263	Up
**216**	3508510	C	T	98.80%	99.32%				SAZ_3263,SAZ_3264	Dn
**217**	3508535	-	T	32.14%	-				SAZ_3263,SAZ_3264	Dn
**218**	3508553	C	G	100%	15%				SAZ_3263,SAZ_3264	Dn
**219**	3508569	G	C	100%	100%				SAZ_3263,SAZ_3264	Dn
**220**	3526694	C	G	100%	98.53%	SAZ_3282	Ns	V262L		
**221**	3539327	T	G	3.15%	-	SAZ_3299	Ns	T680P		
**222**	3547063	T	G	-	3.29%	SAZ_3304	Ns	V488G		
**223**	3559196	T	G	10.13%	-	SAZ_3318	Ns	H42P		
**224**	3569010	A	C	-	7.22%	SAZ_3328	Ns	H202P		
**225**	3569290	G	C	7.87%	-	SAZ_3328	S	P295P		
**226**	3569296	G	C	7.76%	5.56%	SAZ_3328	S	P297P		
**227**	3607011	A	C	3.11%	-	SAZ_3363	Ns	V213G		
**228**	3614856	T	G	-	3.60%				SAZ_3370,SAZ_3371	Dn
**229**	3614861	T	G	3.93%	-				SAZ_3370,SAZ_3371	Dn
**230**	3622775	A	C	-	4.21%	SAZ_3378	Ns	T663P		
**231**	3623408	A	C	3.24%	-	SAZ_3378	Ns	T874P		
**232**	3628030	G	C	7.69%	6.67%	SAZ_3380	Ns	R213P		
**233**	3628039	G	C	6.08%	12.90%	SAZ_3380	Ns	R216P		
**234**	3631842	G	C	18.10%	13.10%				SAZ_3386,SAZ_3385	Up, Dn
**235**	3640547	T	G	4.97%	-				SAZ_3393,SAZ_3394	Up, Dn
**236**	3651736	C	G	3.91%	3.29%	SAZ_3402	Ns	A10P		
**237**	3659892	C	T	3.37%	-				SAZ_3412,SAZ_3413	Dn
**238**	3660590	T	G	-	5.52%	SAZ_3413	Ns	T180P		

**239**	3661216	C	G	6.02%	8.20%				SAZ_3413	Up
**240**	3664997	T	G	3.49%	-	SAZ_3418	Ns	V174G		
**241**	3676800	A	G	98.57%	98.41%				SAZ_3427,SAZ_3426	Up, Dn
**242**	3680271	G	C	5.61%	6.04%				SAZ_3430,SAZ_3429	Up, Dn
**243**	3680278	G	C	10.15%	11.27%				SAZ_3430,SAZ_3429	Up, Dn
**244**	3680281	G	C	5.76%	6.34%				SAZ_3430,SAZ_3429	Up, Dn
**245**	3680294	G	C	7.88%	4.05%				SAZ_3430,SAZ_3429	Up, Dn
**246**	3686020	G	C	3.85%	4.72%				SAZ_3435,SAZ_3434	Up, Dn
**247**	3694482	G	C	100%	100%				SAZ_3440,SAZ_3441	Up, Dn
**248**	3748290	G	-	5%	-	SAZ_3494	FS	T67fs		
**249**	3748490	T	G	6.80%	9.97%	SAZ_3494	Ns	M1L		
**250**	3760667	C	G	4.23%	-	SAZ_3504	S	P50P		
**251**	3765571	T	C	99.55%	99.47%	SAZ_3509	S	Q233Q		
**252**	3789710	G	C	3.46%	3.26%	SAZ_3532	Ns	R34G		
**253**	3838267	T	-	36.49%	-				SAZ_3573,SAZ_3572	Up, Dn
**254**	3846539	T	G	-	5.96%				SAZ_3578,SAZ_3579	Dn
**255**	3857357	T	G	-	3.46%	SAZ_3587	Ns	E19A		
**256**	3869592	T	C	10.64%	9.24%	SAZ_3597	Ns	L421P		
**257**	3869598	T	C	9.25%	9.74%	SAZ_3597	Ns	L423P		
**258**	3869601	G	C	8.36%	7.17%	SAZ_3597	Ns	R424P		
**259**	3869607	T	C	5.88%	5.42%	SAZ_3597	Ns	L426P		
**260**	3872667	G	C	100%	98.33%				SAZ_3601	Dn
**261**	3873609	T	G	5.07%	4.33%	SAZ_3602	Ns	V214G		
**262**	3882105	G	A	50.77%	38.03%	SAZ_3609	S	G106G		
**263**	3882135	G	C	65.85%	62.82%	SAZ_3609	Ns	Q116H		
**264**	3882138	C	T	66.13%	62.65%	SAZ_3609	S	G117G		
**265**	3882156	G	T	78.36%	75.26%	SAZ_3609	S	P123P		
**266**	3882165	G	C	81.38%	79.08%	SAZ_3609	S	P126P		
**267**	3882198	C	A	7.59%	6.60%	SAZ_3609	S	G137G		
**268**	3892351	G	C	4.27%	-	SAZ_3618	Ns	A147G		
**269**	3894107	G	C	8.88%	8.68%	SAZ_3619	S	G153G		
**270**	3894111	G	C	7.03%	5.88%	SAZ_3619	Ns	A152G		
**271**	3894115	G	C	6.37%	5.02%	SAZ_3619	Ns	R151G		
**272**	3894119	G	C	4.80%	3.91%	SAZ_3619	S	R149R		
**273**	3925048	C	T	98.91%	98.56%				SAZ_3645,SAZ_3646	Up

**274**	3925060	C	G	100%	99.60%				SAZ_3645,SAZ_3646	Up
**275**	3925084	C	A	99.42%	98.80%				SAZ_3645,SAZ_3646	Up
**276**	3925089	A	T	99.34%	99.35%				SAZ_3645,SAZ_3646	Up
**277**	3925093	C	A	100%	99.25%				SAZ_3645,SAZ_3646	Up
**278**	3925170	G	C	4.79%	-				SAZ_3646	Up
**279**	3959063	A	C	6.81%	4.48%				SAZ_3668,SAZ_3667	Up, Dn
**280**	4030865	C	G	4.85%	3.79%				SAZ_3727	Dn
**281**	4043008	G	T	100%	100%				SAZ_3738,SAZ_3739	IR
**282**	4043014	G	T	100%	100%				SAZ_3738,SAZ_3739	IR
**283**	4043047	G	T	99.42%	99.39%				SAZ_3738,SAZ_3739	IR
**284**	4043084	A	G	100%	100%				SAZ_3738,SAZ_3739	IR
**285**	4043180	A	T	20%	22.06%				SAZ_3738,SAZ_3739	IR
**286**	4043227	G	T	25.41%	24.87%				SAZ_3738,SAZ_3739	IR
**287**	4043235	C	T	25.99%	28.06%				SAZ_3738,SAZ_3739	IR
**288**	4043304	-	G	26.89%	33.61%				SAZ_3739	Dn
**289**	4043381	A	G	27.47%	26.76%				SAZ_3739	Dn
**290**	4043455	C	G	14.40%	19.49%				SAZ_3739	Dn
**291**	4078994	T	G	3.12%	-				SAZ_3778,SAZ_3777	Up, Dn
**292**	4083090	C	T	99.25%	99.64%	SAZ_3781	Ns	P43L		
**293**	4084423	A	T	46.81%	41.19%	SAZ_3783	Ns	V481E		
**294**	4133316	G	C	10.75%	12.43%	SAZ_3832	Ns	R61G		
**295**	4133385	G	C	4.67%	4.90%	SAZ_3832	Ns	R38G		
**296**	4134391	A	C	3.49%	3.21%	SAZ_3833	Ns	T308P		
**297**	4138640	C	A	8.07%	4.61%				SAZ_3836	Dn
**298**	4138646	G	A	4.71%	5.48%				SAZ_3836	Dn
**299**	4156743	T	G	-	3.91%	SAZ_3850,SAZ_385[illegible]	Ns	N45T		
**300**	4161326	C	G	6.30%	3.57%	SAZ_3860	S	G74G		
**301**	4161329	C	G	10.24%	9.50%	SAZ_3860	S	G75G		
**302**	4161336	C	G	11.67%	12.96%	SAZ_3860	Ns	R78G		
**303**	4161341	C	G	9.52%	11.46%	SAZ_3860	S	G79G		
**304**	4167729	C	-	57.45%	65%				SAZ_3862,SAZ_3863	Dn
**305**	4167743	-	T	13.75%	17.50%				SAZ_3862,SAZ_3863	Dn
**306**	4168364	G	C	32.81%	34%	SAZ_3863	Ns	R364G		
**307**	4168367	A	C	15.25%	-	SAZ_3863	Ns	W363G		
**308**	4168371	G	C	24.68%	28.81%	SAZ_3863	S	G361G		

**309**	4168377	G	C	15.28%	15.62%	SAZ_3863	S	G359G		
**310**	4183545	G	T	-	7.49%	SAZ_3874	Ns	P1341T		
**311**	4198141	C	A	99.01%	97.67%	SAZ_3885	Ns	G25V		
**312**	4198147	C	A	98.67%	96.77%	SAZ_3885	Ns	G23V		
**313**	4211245	A	-	93.66%	93.20%				SAZ_3895,SAZ_3896	IR
**314**	4211394	TGTTCG	-	79.19%	77.44%				SAZ_3895,SAZ_3896	IR
**315**	4220198	-	A	5.29%	-				SAZ_3896,SAZ_3897	IR
**316**	4220236	A	G	12.66%	-				SAZ_3896,SAZ_3897	IR
**317**	4238490	G	A	-	5.53%				SAZ_3911	Up
**318**	4272356	T	G	-	3.34%				SAZ_3945	Dn
**319**	4288943	C	G	4.68%	4%				SAZ_3961,SAZ_3962	Up, Dn
**320**	4315439	G	T	99.24%	100%	SAZ_3986	Ns	R54M		
**321**	4315453	G	T	99.61%	99.12%	SAZ_3986	Ns	A59S		
**322**	4315475	G	A	98.65%	99.51%	SAZ_3986	Ns	R66H		
**323**	4315498	C	A	100%	100%	SAZ_3986	Ns	L74I		
**324**	4315520	C	T	97.35%	98.45%	SAZ_3986	Ns	T81I		
**325**	4315537	C	A	100%	-	SAZ_3986	Ns	P87T		
**326**	4315543	A	C	100%	100%	SAZ_3986	Ns	M89L		
**327**	4315546	C	A	100%	-	SAZ_3986	Ns	P90T		
**328**	4315547	C	A	100%	-	SAZ_3986	Ns	P90Q		
**329**	4319510	C	G	-	4.18%				SAZ_3989,SAZ_3990	Dn
**330**	4348859	G	C	9.36%	10.12%	SAZ_4016	S	G32G		
**331**	4348872	T	C	12.59%	16.15%	SAZ_4016	Ns	W37R		
**332**	4348968	G	A	-	3.07%	SAZ_4016	Ns	V69I		
**333**	4348995	A	C	-	3.63%	SAZ_4016	S	R78R		
**334**	4349097	T	C	3.37%	-	SAZ_4016	Ns	W112R		
**335**	4349108	C	A	3.40%	-	SAZ_4016	S	S115S		
**336**	4349229	A	C	-	3.47%				SAZ_4016,SAZ_4017	Dn
**337**	4368223	C	A	99.04%	100%				SAZ_4030	Dn
**338**	4368226	C	A	98.95%	100%				SAZ_4030	Dn
**339**	4368231	G	-	6.14%	10.26%				SAZ_4030	Dn
**340**	4368257	C	-	75.53%	78.16%				SAZ_4030	Dn
**341**	4368269	C	T	100%	100%				SAZ_4030	Dn
**342**	4388418	A	C	-	3.95%				SAZ_4046	Up
**343**	4389340	G	C	-	5.37%	SAZ_4046	S	P230P		

**344**	4405089	-	CAGC	-	3.40%				SAZ_4062,SAZ_4063	Up, Dn
**345**	4422002	G	T	47.61%	41.42%				SAZ_4077,SAZ_4078	IR
**346**	4432377	A	C	4.37%	6.51%				SAZ_4093,SAZ_4094	Up, Dn
**347**	4432382	G	C	9.77%	12.93%				SAZ_4093,SAZ_4094	Up, Dn
**348**	4432388	G	C	7.52%	8.16%				SAZ_4093,SAZ_4094	Up, Dn
**349**	4432393	G	C	-	3.92%				SAZ_4093,SAZ_4094	Up, Dn
**350**	4439509	T	C	-	5.05%	SAZ_4102	Ns	T623A		
**351**	4442880	A	C	-	3.16%	SAZ_4103	Ns	T420P		
**352**	4446996	C	A	99.46%	98.54%				SAZ_4107,SAZ_4108	IR
**353**	4447003	C	A	99.02%	100%				SAZ_4107,SAZ_4108	IR
**354**	4447041	G	T	100%	98.51%				SAZ_4108	Up
**355**	4447048	C	A	97.79%	99.04%				SAZ_4108	Up
**356**	4448580	T	G	-	10.11%				SAZ_4109,SAZ_4110	IR
**357**	4448586	T	G	9.76%	7.95%				SAZ_4109,SAZ_4110	IR
**358**	4448593	T	G	-	6.25%				SAZ_4109,SAZ_4110	IR
**359**	4448598	T	G	-	8.33%				SAZ_4109,SAZ_4110	IR
**360**	4450930	T	G	-	6.48%	SAZ_4111	Ns	V364G		
**361**	4450933	C	G	3.60%	4%	SAZ_4111	Ns	A365G		
**362**	4450937	C	G	4.27%	6.13%	SAZ_4111	S	G366G		
**363**	4451285	C	G	10.08%	10.88%				SAZ_4111,SAZ_4112	Dn
**364**	4456220	A	C	-	3.25%				SAZ_4115,SAZ_4116	Dn
**365**	4461545	A	C	9.84%	7.69%				SAZ_4120,SAZ_4119	Up, Dn
**366**	4461551	A	C	5.41%	8.67%				SAZ_4120,SAZ_4119	Up, Dn
**367**	4474705	G	A	3.98%	-	SAZ_4132	Ns	A132V		
**368**	4484704	A	C	-	3.51%	SAZ_4143	S	G119G		
**369**	4512619	G	C	4.26%	6.55%				SAZ_4171,SAZ_4170	Up, Dn
**370**	4512633	-	CGAAA	12.02%	-				SAZ_4171,SAZ_4170	Up, Dn
**371**	4512638	-	GT	6.86%	4.89%				SAZ_4171,SAZ_4170	Up, Dn
**372**	4523380	T	G	-	8.14%				SAZ_4178,SAZ_4179	Dn
**373**	4523390	T	G	16.67%	-				SAZ_4178,SAZ_4179	Dn
**374**	4523395	C	G	16.87%	-				SAZ_4178,SAZ_4179	Dn
**375**	4564295	G	C	6.63%	5.61%				SAZ_4216,SAZ_4215	Up, Dn
**376**	4564299	G	C	4.83%	4.71%				SAZ_4216,SAZ_4215	Up, Dn
**377**	4565125	C	T	-	5.25%	SAZ_4216	S	V267V		
**378**	4566051	A	C	-	3.49%	SAZ_4218	Ns	W144G		

**379**	4566794	T	G	81.82%	80.46%				SAZ_4219	Up
**380**	4566820	G	T	100%	100%				SAZ_4219	Up
**381**	4571147	A	C	5.43%	-				SAZ_4222,SAZ_4223	Up, Dn
**382**	4571153	G	C	6.80%	5.38%				SAZ_4222,SAZ_4223	Up, Dn
**383**	4615553	A	C	3.41%	6.17%	SAZ_4262	StopLoss	*330S		
**384**	4615557	C	A	39.91%	50%				SAZ_4262,SAZ_4263	Dn
**385**	4615562	C	G	5.83%	-				SAZ_4262,SAZ_4263	Dn
**386**	4615564	A	C	3.81%	3.81%				SAZ_4262,SAZ_4263	Dn
**387**	4623190	A	C	3.15%	-	SAZ_4270	Ns	M1R		
**388**	4678173	C	G	22.73%	10.67%	SAZ_4336	Ns	A553P		
**389**	4692608	-	G	17.48%	20%				SAZ_4348	UTR5
**390**	4743050	C	T	-	3.26%				SAZ_4400	Dn
**391**	4744698	G	A	4.85%	3.07%				SAZ_4401,SAZ_4402	IR
**392**	4749899	T	G	3.31%	3.93%				SAZ_4405,SAZ_4406	Dn
**393**	4758240	G	-	-	4.38%				SAZ_4412,SAZ_4413	Up
**394**	4779815	T	G	-	3.11%	SAZ_4432	Ns	T21P		
**395**	4793112	A	C	-	3.68%				SAZ_4445,SAZ_4446	Up, Dn
**396**	4793120	A	C	5.10%	5.75%				SAZ_4445,SAZ_4446	Up, Dn
**397**	4793567	C	T	34.39%	29.67%	SAZ_4446	S	L272L		
**398**	4796460	A	-	92.90%	92.45%				SAZ_4448	Up
**399**	4796482	T	C	99.54%	99.62%				SAZ_4448	Up
**400**	4796609	TGTTCG	-	68.99%	63.92%				SAZ_4448	Up
**401**	4830172	A	C	3.12%	4.65%	SAZ_4470	Ns	V223G		
**402**	4834424	T	G	5.17%	5.56%	SAZ_4475	Ns	T74P		
**403**	4857455	C	G	3.76%	3.35%	SAZ_4495	S	A20A		
**404**	4894220	C	G	-	4.62%	SAZ_4528	Ns	A882P		
**405**	4900369	C	G	-	6.69%	SAZ_4534	Ns	G205A		
**406**	4906277	C	T	99.38%	99.42%				SAZ_4537	Dn
**407**	4906288	C	A	99.53%	100%				SAZ_4537	Dn
**408**	4906316	G	T	98.38%	100%				SAZ_4537	Dn
**409**	4906325	G	T	98.75%	99.35%				SAZ_4537	Dn
**410**	4906519	G	C	99.68%	99.73%				SAZ_4538	Up
**411**	4937034	C	G	5.57%	4.74%	SAZ_4571	Ns	G584R		
**412**	4937039	C	G	3.75%	3.80%	SAZ_4571	Ns	R582P		
**413**	4937044	C	G	7.71%	6.59%	SAZ_4571	S	P580P		

**414**	4937052	T	G	-	3.73%	SAZ_4571	Ns	T578P		
**415**	4942967	T	G	3.10%	-	SAZ_4578	Ns	Y40D		
**416**	4955156	G	C	3.37%	5.86%	SAZ_4593	Ns	R29P		
**417**	4955160	G	C	-	3.31%	SAZ_4593	S	P30P		
**418**	4974877	C	G	-	10.84%				SAZ_4609,SAZ_4610	Up, Dn
**419**	4978962	T	G	3.59%	5.08%	SAZ_4616	Ns	V71G		
**420**	5059134	A	C	7.01%	3.40%	SAZ_4686	Ns	V58G		
**421**	5065540	G	C	4.28%	5.73%	SAZ_4693	Ns	A6P		
**422**	5090774	T	G	-	3.78%	SAZ_4715	Ns	T238P		
**423**	5091855	A	C	3.09%	3.14%	SAZ_4716	Ns	V95G		
**424**	5092877	C	G	-	6.67%	SAZ_4718	S	P69P		
**425**	5092882	C	G	7.14%	12.71%	SAZ_4718	Ns	A68P		
**426**	5092885	T	G	7.41%	7.46%	SAZ_4718	Ns	T67P		
**427**	5092897	C	G	4.88%	-	SAZ_4718	Ns	A63P		
**428**	5097505	-	G	3.70%	4.87%				SAZ_4724	Dn
**429**	5099653	C	A	98.70%	100%	SAZ_4726	Ns	P362T		
**430**	5124410	A	C	3.53%	6.60%				SAZ_4748,SAZ_4749	Dn
**431**	5212135	A	G	6.54%	3.35%				SAZ_4827,SAZ_4828	Up, Dn
**432**	5212141	C	G	5.74%	4.87%				SAZ_4827,SAZ_4828	Up, Dn
**433**	5212147	C	G	4.76%	3.90%				SAZ_4827,SAZ_4828	Up, Dn
**434**	5212151	A	G	7.92%	4.74%				SAZ_4827,SAZ_4828	Up, Dn
**435**	5212157	A	G	-	3.45%				SAZ_4827,SAZ_4828	Up, Dn
**436**	5238716	G	C	-	7.09%				SAZ_4856,SAZ_4855	Up, Dn
**437**	5238720	G	C	4.49%	7.04%				SAZ_4856,SAZ_4855	Up, Dn
**438**	5238730	G	C	-	3.72%				SAZ_4856,SAZ_4855	Up, Dn
**439**	5259624	C	G	11.65%	12.64%	SAZ_4874	S	G310G		
**440**	5261377	C	G	9.20%	-	SAZ_4876	S	P54P		
**441**	5261382	C	G	17.65%	12.99%	SAZ_4876	Ns	A53P		
**442**	5261637	T	G	5.04%	4.15%				SAZ_4876	Up
**443**	5293162	C	G	-	6.11%	SAZ_4904	S	G300G		
**444**	5301993	G	T	3.06%	-	SAZ_4911	Ns	G188W		
**445**	5302002	C	G	14.16%	10%	SAZ_4911	Ns	L191V		
**446**	5319634	T	G	5.62%	-	SAZ_4931	S	P153P		
**447**	5319639	C	G	4.66%	4.42%	SAZ_4931	Ns	A152P		
**448**	5319643	C	G	-	4.49%	SAZ_4931	S	P150P		

**449**	5319648	C	G	7.69%	7.95%	SAZ_4931	Ns	E149Q		
**450**	5322272	T	C	4.49%	-				SAZ_4933,SAZ_4934	Up, Dn
**451**	5357670	C	T	49.38%	35.73%	SAZ_4975	Ns	A235V		
**452**	5373421	C	G	-	3.45%				SAZ_4989,SAZ_4990	Up, Dn
**453**	5373425	C	G	3.59%	4.09%				SAZ_4989,SAZ_4990	Up, Dn
**454**	5399901	A	C	4.17%	-	SAZ_5014	Ns	V294G		
**455**	5440405	A	C	9.09%	-				SAZ_5050,SAZ_5051	Dn
**456**	5506175	T	G	-	9.20%				SAZ_5103	Up
**457**	5506178	T	G	-	8.49%				SAZ_5103	Up
**458**	5506187	T	G	7.29%	7.22%				SAZ_5103	Up
**459**	5506193	C	G	6.09%	6.84%				SAZ_5103	Up
**460**	5534991	T	C	8.55%	-				SAZ_5140	Dn
**461**	5535010	-	C	6.52%	-				SAZ_5140,SAZ_5141	IR
**462**	5535758	-	G	9.55%	9.02%				SAZ_5141	Dn
**463**	5535982	G	T	8.46%	10.09%				SAZ_5141,SAZ_5142	IR
**464**	5562278	T	G	98.33%	99.17%				SAZ_5166,SAZ_5167	Dn
**465**	5562283	T	G	100%	100%				SAZ_5166,SAZ_5167	Dn
**466**	5562311	C	G	99.41%	99.46%				SAZ_5166,SAZ_5167	Dn
**467**	5562319	C	G	98.56%	99.56%				SAZ_5166,SAZ_5167	Dn
**468**	5584341	C	G	9.09%	6.60%	SAZ_5188	Ns	A243P		
**469**	5594749	C	T	-	16.08%	SAZ_5199	Ns	E152K		
**470**	5615628	G	A	45.24%	43.62%	SAZ_5219	S	L204L		
**471**	5622375	C	A	98.28%	-				SAZ_5225	Up
**472**	5629416	ACGAAA	-	84.14%	86.71%				SAZ_5228	Dn
**473**	5657433	T	C	3.35%	-				SAZ_5253,SAZ_5252	Up, Dn
**474**	5657517	T	C	-	4.26%				SAZ_5253	Up
**475**	5657528	G	A	-	4.65%				SAZ_5253	Up
**476**	5657552	T	A	3.34%	6.84%				SAZ_5253	Up
**477**	5657738	A	-	4.38%	8.52%				SAZ_5253,SAZ_5254	IR
**478**	5657742	CGCC	-	4.32%	8.03%				SAZ_5253,SAZ_5254	IR
**479**	5657772	A	C	3.24%	6.96%				SAZ_5253,SAZ_5254	IR
**480**	5657779	A	G	-	4.80%				SAZ_5253,SAZ_5254	IR
**481**	5680873	C	G	100%	100%	SAZ_5280,SAZ_5281	Ns	P68A		
**482**	5680891	-	T	21.33%	31.43%	SAZ_5280,SAZ_5281	FS	A74fs		
**483**	5689051	A	C	3.19%	-	SAZ_5290	Ns	V144G		

**484**	5777139	C	G	3.85%	-				SAZ_5392,SAZ_5393	Up
**485**	5799103	G	C	8.99%	11.90%				SAZ_5416,SAZ_5415	Up, Dn
**486**	5799107	G	C	8%	6.80%				SAZ_5416,SAZ_5415	Up, Dn
**487**	5799116	G	C	5.45%	6.23%				SAZ_5416,SAZ_5415	Up, Dn
**488**	5804556	G	C	6.63%	6.25%				SAZ_5422	Up
**489**	5807226	C	G	9.09%	11.48%	SAZ_5423	Ns	A123P		
**490**	5837662	C	G	99.48%	99.52%	SAZ_5452	Ns	V327L		
**491**	5840154	A	C	-	6.76%	SAZ_5454	S	P19P		
**492**	5866641	G	T	-	100%	SAZ_5477	Ns	G73V		
**493**	5867925	C	T	4.86%	-	SAZ_5477	Ns	P501L		
**494**	5868749	A	C	4.98%	6.72%	SAZ_5477	Ns	T776P		
**495**	5868752	G	C	4.03%	7.38%	SAZ_5477	Ns	A777P		
**496**	5875236	G	A	5.95%	-	SAZ_5482	Ns	V244M		
**497**	5884232	C	T	98.92%	100%				SAZ_5488	Dn
**498**	5884250	C	T	99.17%	100%				SAZ_5488	Dn
**499**	5884256	-	C	17.65%	-				SAZ_5488	Dn
**500**	5884269	-	G	85%	80%				SAZ_5488	Dn
**501**	5884299	A	C	100%	98.39%				SAZ_5488	Dn
**502**	5884318	G	T	97.75%	99.09%				SAZ_5488	Dn
**503**	5884335	G	T	97.44%	99.31%				SAZ_5488	Dn
**504**	5884353	C	T	99.19%	98.67%				SAZ_5488	Dn
**505**	5884375	C	G	100%	100%				SAZ_5488	Dn
**506**	5884394	C	G	100%	100%				SAZ_5488	Dn
**507**	5884436	C	G	100%	-				SAZ_5488	Dn
**508**	5884455	G	A	98.75%	100%				SAZ_5488,SAZ_5489	IR
**509**	5884466	T	G	100%	100%				SAZ_5488,SAZ_5489	IR
**510**	5884480	A	T	100%	100%				SAZ_5488,SAZ_5489	IR
**511**	5884498	C	A	100%	100%				SAZ_5488,SAZ_5489	IR
**512**	5914451	G	C	4.91%	9.59%	SAZ_5514	Ns	A329G		
**513**	5914457	G	C	11.38%	14.71%	SAZ_5514	Ns	A327G		
**514**	5938589	C	G	-	3.24%	SAZ_5548	Ns	A18P		
**515**	5939271	T	G	-	4.25%	SAZ_5549	Ns	Y142S		
**516**	5969967	C	-	16.33%	15.07%	SAZ_5588	FS	H30fs		
**517**	5974382	C	G	-	4.02%				SAZ_5592,SAZ_5593	Dn
**518**	5978420	C	G	-	3.13%	SAZ_5600	S	P461P		

**519**	5995123	A	G	99.29%	100%	SAZ_5611	Ns	D39G		
**520**	5995157	A	G	98.67%	100%	SAZ_5611	S	V50V		
**521**	5995192	G	C	99.60%	100%				SAZ_5610,SAZ_5612,SAZ_5611	Up, Dn
**522**	6015955	C	G	6.71%	7.35%	SAZ_5625	Ns	R158P		
**523**	6037597	T	G	7.80%	-				SAZ_5640	Up
**524**	6037601	C	G	13.72%	7.74%				SAZ_5640	Up
**525**	6040575	T	G	-	5.15%	SAZ_5643	Ns	H296P		
**526**	6075469	A	C	3.31%	-				SAZ_5678,SAZ_5677	Up, Dn
**527**	6081969	A	C	-	3.10%				SAZ_5683	Up
**528**	6096113	C	G	-	3.35%	SAZ_5699	Ns	R20P		
**529**	6110223	T	G	-	10%	SAZ_5716	Ns	T429P		
**530**	6112240	C	G	15.79%	-				SAZ_5717,SAZ_5718	Dn
**531**	6112254	T	C	98.90%	100%				SAZ_5717,SAZ_5718	Dn
**532**	6118655	-	C	-	3.11%				SAZ_5723,SAZ_5722	Up, Dn
**533**	6118660	-	C	4.55%	-				SAZ_5723,SAZ_5722	Up, Dn
**534**	6118664	G	C	9.44%	12.10%				SAZ_5723,SAZ_5722	Up, Dn
**535**	6118669	A	C	-	9.38%				SAZ_5723,SAZ_5722	Up, Dn
**536**	6118672	G	C	5.94%	-				SAZ_5723,SAZ_5722	Up, Dn
**537**	6118683	A	C	5.68%	-				SAZ_5723,SAZ_5722	Up, Dn
**538**	6118687	G	C	3.32%	6.67%				SAZ_5723,SAZ_5722	Up, Dn
**539**	6123831	C	T	3.37%	-	SAZ_5727	S	D5D		
**540**	6132075	G	C	3.14%	4.02%	SAZ_5733	S	P297P		
**541**	6132082	G	C	7.41%	5.38%	SAZ_5733	Ns	A300P		
**542**	6132092	G	C	6.36%	3.93%	SAZ_5733	Ns	R303P		
**543**	6181373	T	G	6.48%	-				SAZ_5784,SAZ_5785	Up, Dn
**544**	6184040	A	C	-	4.14%	SAZ_5787	Ns	Y89D		
**545**	6184794	A	C	-	3.59%	SAZ_5788	Ns	V1G		
**546**	6197678	C	G	87.10%	80.60%				SAZ_5800,SAZ_5799	Up, Dn
**547**	6220753	G	C	6.62%	6.06%	SAZ_5821	Ns	A9P		
**548**	6224397	A	C	3.55%	-				SAZ_5824	Dn
**549**	6225730	G	C	4.62%	-	SAZ_5826	Ns	A188P		
**550**	6225854	G	C	-	4.43%				SAZ_5827,SAZ_5826	Up, Dn
**551**	6261737	G	C	100%	100%	SAZ_5860,SAZ_5861	Ns	D278E		
**552**	6261741	C	T	98.53%	99.30%	SAZ_5860,SAZ_5861	Ns	R277H		
**553**	6261754	T	G	100%	99.60%	SAZ_5861	Ns	N273H		

**554**	6261774	C	T	100%	99.45%	SAZ_5861	Ns	G266E		
**555**	6261795	T	A	98.26%	98.88%	SAZ_5861	Ns	E259V		
**556**	6261815	C	A	100%	99.50%	SAZ_5861	S	R252R		
**557**	6261825	A	C	100%	100%	SAZ_5861	Ns	L249R		
**558**	6261845	A	G	100%	99.42%	SAZ_5861	S	P242P		
**559**	6261872	C	T	100%	99.12%	SAZ_5861	S	E233E		
**560**	6289616	C	G	100%	100%	SAZ_5888	Ns	K313N		
**561**	6289634	G	T	99.10%	98.53%	SAZ_5888	S	A307A		
**562**	6289649	G	T	99.59%	100%	SAZ_5888	Ns	D302E		
**563**	6289659	G	T	99.13%	100%	SAZ_5888	Ns	P299Q		
**564**	6289684	G	T	98.54%	99.40%	SAZ_5888	Ns	Q291K		
**565**	6290534	C	A	98.67%	99.49%	SAZ_5888	S	R7R		
**566**	6290559	G	C	100%	100%				SAZ_5888,SAZ_5889	Up, Dn
**567**	6315093	C	G	3.74%	6.50%	SAZ_5906	Ns	A139P		
**568**	6391533	A	C	3.41%	4.08%				SAZ_5984,SAZ_5985	Dn
**569**	6391537	A	G	16.87%	15%				SAZ_5984,SAZ_5985	Dn
**570**	6391543	C	G	29.14%	24.36%				SAZ_5984,SAZ_5985	Dn
**571**	6414293	C	G	4.35%	4.66%				SAZ_6006,SAZ_6007	Up, Dn
**572**	6418061	T	G	3.02%	-	SAZ_6010	Ns	V285G		
**573**	6420580	T	G	3.46%	-	SAZ_6013	Ns	V35G		
**574**	6429425	G	C	4.80%	6.54%	SAZ_6022	Ns	R397P		
**575**	6429431	T	G	-	3.26%	SAZ_6022	Ns	V399G		
**576**	6434872	T	G	-	4.06%	SAZ_6026	Ns	Y13S		
**577**	6475217	T	G	-	6.25%	SAZ_6066	Ns	T41P		
**578**	6489349	A	C	3.43%	-				SAZ_6082,SAZ_6083	Up, Dn
**579**	6520942	G	C	3.28%	-	SAZ_6109	S	G28G		
**580**	6525330	C	G	5.90%	7.18%				SAZ_6112	Up
**581**	6525337	T	G	4.02%	8.54%				SAZ_6112	Up
**582**	6525341	C	G	10.61%	13.78%				SAZ_6112	Up
**583**	6546812	G	C	100%	100%	SAZ_6132	Ns	G196A		
**584**	6557281	A	G	98.95%	100%	SAZ_6140	Ns	S971G		
**585**	6561453	T	C	6.12%	-	SAZ_6141	S	L947L		
**586**	6561457	T	-	7.36%	-	SAZ_6141	FS	V948fs		
**587**	6561463	-	TGCTGGTGCTGGTGCTGG	17.39%	15.79%	SAZ_6141	nonFS	S950delinsSAGAGAG		
**588**	6564716	T	C	98.97%	100%	SAZ_6141	S	I2034I		

**589**	6623163	G	C	13.27%	12.28%				SAZ_6153,SAZ_6154	Dn
**590**	6629233	G	C	23.81%	-				SAZ_6158,SAZ_6159	Dn
**591**	6629237	G	C	13.14%	14.60%				SAZ_6158,SAZ_6159	Dn
**592**	6629240	G	C	5.92%	5.41%				SAZ_6158,SAZ_6159	Dn
**593**	6634197	G	C	100%	100%				SAZ_6165,SAZ_6164	Up, Dn
**594**	6635816	C	T	3.39%	4%	SAZ_6165	Ns	A456V		
**595**	6637563	T	G	-	3.63%	SAZ_6166	Ns	Y126S		
**596**	6639790	C	-	3.11%	4.17%				SAZ_6168,SAZ_6169	Up
**597**	6669166	T	G	-	4.18%	SAZ_6197	Ns	T59P		
**598**	6674070	-	GG	11.70%	-	SAZ_6202	FS	T88fs		
**599**	6674330	AT	-	92.86%	-	SAZ_6202	FS			
**600**	6729865	C	G	5.46%	3.83%				SAZ_6247,SAZ_6248	Up, Dn
**601**	6729870	C	G	3.89%	-				SAZ_6247,SAZ_6248	Up, Dn
**602**	6731257	C	G	-	5.49%				SAZ_6248,SAZ_6249	Up, Dn
**603**	6731261	C	G	5.33%	7.95%				SAZ_6248,SAZ_6249	Up, Dn
**604**	6746269	C	G	11.74%	11.71%				SAZ_6262,SAZ_6261	Up, Dn
**605**	6799850	A	C	3.11%	-				SAZ_6305,SAZ_6304	Up, Dn
**606**	6818984	A	C	4.47%	-	SAZ_6320	Ns	T655P		
**607**	6818993	A	C	4.40%	-	SAZ_6320	Ns	T658P		
**608**	6842861	C	G	100%	100%				SAZ_6341,SAZ_6342	IR
**609**	6870050	C	G	100%	99.25%	SAZ_6371	Ns	P809A		
**610**	6870096	C	A	100%	99.30%	SAZ_6371	Ns	A824E		
**611**	6870130	G	C	100%	100%	SAZ_6371	S	A835A		
**612**	6897161	C	G	-	3.54%				SAZ_6396,SAZ_6395	Up, Dn
**613**	6915646	A	C	8.45%	-				SAZ_6413,SAZ_6414	Dn
**614**	6915658	A	C	16.46%	-				SAZ_6413,SAZ_6414	Dn
**615**	6916361	T	G	-	3.14%	SAZ_6414	Ns	T211P		
**616**	6916929	C	G	4.86%	-	SAZ_6414	S	P21P		
**617**	6916935	C	G	4.83%	5.56%	SAZ_6414	S	P19P		
**618**	6939194	A	C	-	3.67%	SAZ_6435	Ns	T183P		
**619**	6972471	A	C	-	5.66%				SAZ_6465,SAZ_6466	Dn
**620**	6986067	A	G	3.66%	-	SAZ_6481	Ns	L270P		
**621**	7006692	C	T	11.41%	6.54%				SAZ_6501	Dn
**622**	7006694	-	TCTT	3.45%	-				SAZ_6501	Dn
**623**	7006695	GC	-	-	3.20%				SAZ_6501	Dn

**624**	7006744	C	T	4.55%	-				SAZ_6501	Dn
**625**	7006747	A	-	10.62%	7.04%				SAZ_6501	Dn
**626**	7006752	C	-	9.88%	7.59%				SAZ_6501	Dn
**627**	7008653	T	C	100%	100%				SAZ_6503,SAZ_6504	Dn
**628**	7027276	G	T	99.24%	98.90%				SAZ_6517,SAZ_6518	IR
**629**	7027400	T	C	3.56%	3.12%				SAZ_6517,SAZ_6518	IR
**630**	7027401	G	A	3.34%	-				SAZ_6517,SAZ_6518	IR
**631**	7027413	A	G	10.65%	3.80%				SAZ_6517,SAZ_6518	IR
**632**	7027414	C	T	4.99%	3.02%				SAZ_6517,SAZ_6518	IR
**633**	7027437	G	C	6.58%	5.45%				SAZ_6517,SAZ_6518	IR
**634**	7027438	T	A	-	7.13%				SAZ_6517,SAZ_6518	IR
**635**	7027446	G	A	14.23%	13.23%				SAZ_6517,SAZ_6518	IR
**636**	7027448	G	C	17.20%	19.29%				SAZ_6517,SAZ_6518	IR
**637**	7027477	A	G	9.78%	8.42%				SAZ_6517,SAZ_6518	IR
**638**	7027535	C	T	47.40%	40.62%				SAZ_6517,SAZ_6518	IR
**639**	7027602	-	C	29%	37.78%				SAZ_6517,SAZ_6518	IR
**640**	7033347	ACGAAA	-	90.86%	88.74%				SAZ_6519	Up
**641**	7033414	T	A	100%	99.55%				SAZ_6519	Up
**642**	7038295	A	G	-	16.42%				SAZ_6523	Dn
**643**	7040505	G	C	-	8.24%	SAZ_6525	Ns	A282P		
**644**	7041774	C	G	4.07%	3.61%				SAZ_6526	Dn
**645**	7041779	C	G	3.35%	-				SAZ_6526	Dn
**646**	7041787	T	G	7.33%	8.04%				SAZ_6526	Dn
**647**	7087958	A	C	-	4.17%	SAZ_6560	Ns	T786P		
**648**	7102722	A	G	-	4.70%	SAZ_6565	Ns	L23P		
**649**	7171176	C	G	4.91%	-	SAZ_6619	Ns	A35P		
**650**	7175172	A	C	3.30%	-	SAZ_6624	Ns	V210G		
**651**	7203843	-	C	-	4.14%	SAZ_6654	FS	D241fs		
**652**	7217853	C	A	20.90%	15.22%	SAZ_6666	S	T188T		
**653**	7234060	C	T	-	5.03%	SAZ_6676	S	A79A		
**654**	7246834	T	G	-	3.03%	SAZ_6691	Ns	L78V		
**655**	7250449	T	G	90%	-	SAZ_6695	Ns	S328A		
**656**	7250452	-	A	39.22%	-	SAZ_6695	FS	A329fs		
**657**	7282614	A	C	3.63%	4.88%	SAZ_6723	S	P190P		
**658**	7299628	C	T	3.04%	-	SAZ_6739	StopGain	W210*		

**659**	7325399	A	C	3.60%	-	SAZ_6760	S	G396G		
**660**	7327972	T	G	-	6.84%	SAZ_6762	Ns	L78R		
**661**	7365869	C	G	5.64%	3.92%	SAZ_6806	Ns	R27G		
**662**	7370150	C	G	10.46%	6.36%	SAZ_6808	Ns	R45P		
**663**	7373400	G	A	3.29%	-				SAZ_6812,SAZ_6813	Up
**664**	7378839	A	G	3.83%	4.73%	SAZ_6817	S	P8P		
**665**	7383349	T	G	13.40%	-				SAZ_6824,SAZ_6823	Up, Dn
**666**	7423470	C	T	3.94%	-	SAZ_6873	Ns	R43C		
**667**	7431270	A	G	6.52%	11.76%	SAZ_6880	S	D97D		
**668**	7431280	A	G	5.80%	11.65%	SAZ_6880	Ns	V94A		
**669**	7451950	A	C	4.63%	4.96%				SAZ_6907	Up
**670**	7455276	C	A	98.53%	99.09%	SAZ_6911	S	T27T		
**671**	7455315	T	C	98.72%	100%	SAZ_6911	S	G40G		
**672**	7455332	C	A	100%	100%	SAZ_6911	Ns	A46D		
**673**	7455353	T	C	98.96%	100%	SAZ_6911	Ns	V53A		
**674**	7455857	C	G	100%	100%	SAZ_6911	Ns	S221C		
**675**	7455877	A	G	100%	100%	SAZ_6911	Ns	S228G		
**676**	7455899	A	G	100%	99.01%	SAZ_6911	Ns	H235R		
**677**	7455919	G	C	100%	100%	SAZ_6911	Ns	A242P		
**678**	7455937	C	A	100%	99.28%	SAZ_6911	S	R248R		
**679**	7455950	G	A	100%	100%	SAZ_6911	Ns	G252D		
**680**	7456417	C	T	-	7.01%	SAZ_6911,SAZ_6912	Ns	A352T		
**681**	7468839	T	G	3.02%	-	SAZ_6927	Ns	T189P		
**682**	7491151	T	G	5.73%	-	SAZ_6952	Ns	T63P		
**683**	7492952	C	G	3.65%	-	SAZ_6954	S	P446P		
**684**	7512773	C	T	-	3.95%	SAZ_6979	S	V337V		
**685**	7564186	T	G	-	3.52%	SAZ_7036	S	A33A		
**686**	7585440	T	G	3.25%	-	SAZ_7056	Ns	T158P		
**687**	7587102	A	C	-	3.12%				SAZ_7057,SAZ_7058	Up
**688**	7598508	C	T	4%	-	SAZ_7068	S	G46G		
**689**	7633377	A	G	-	100%				SAZ_7099	Up
**690**	7653512	C	G	100%	99.48%				SAZ_7117	Up
**691**	7653524	C	A	99.24%	99.45%				SAZ_7117	Up
**692**	7653544	C	T	99.59%	98.29%				SAZ_7117	Up
**693**	7653565	G	T	97.54%	97.78%				SAZ_7117,SAZ_7118	Up

**694**	7653569	G	T	99.19%	97.85%				SAZ_7117,SAZ_7118	Up
**695**	7653588	A	C	98.55%	97.98%				SAZ_7117,SAZ_7118	Up
**696**	7653595	A	C	100%	98.90%				SAZ_7117,SAZ_7118	Up
**697**	7653606	T	C	100%	100%				SAZ_7117,SAZ_7118	Up
**698**	7653615	G	C	98.28%	-				SAZ_7117,SAZ_7118	Up
**699**	7653625	T	C	98.67%	98.08%				SAZ_7117,SAZ_7118	Up
**700**	7653632	A	C	98.59%	100%				SAZ_7117,SAZ_7118	Up
**701**	7653636	C	T	100%	40%				SAZ_7117,SAZ_7118	Up
**702**	7653640	C	A	98.33%	-				SAZ_7117,SAZ_7118	Up
**703**	7653660	T	G	100%	-				SAZ_7117,SAZ_7118	Up
**704**	7653664	C	G	100%	-				SAZ_7117,SAZ_7118	Up
**705**	7653691	G	T	100%	98.18%				SAZ_7117,SAZ_7118	Up
**706**	7653717	G	T	100%	98.53%				SAZ_7117,SAZ_7118	Up
**707**	7653722	G	T	100%	95.38%				SAZ_7117,SAZ_7118	Up
**708**	7653748	C	A	100%	98.65%				SAZ_7117,SAZ_7118	Up
**709**	7653768	T	G	98.44%	100%				SAZ_7118	Up
**710**	7653781	A	G	98.53%	100%				SAZ_7118	Up
**711**	7662348	T	G	-	3.43%	SAZ_7125	S	G433G		
**712**	7670663	A	C	-	5.10%	SAZ_7134	Ns	D314A		
**713**	7698581	C	G	3.21%	-	SAZ_7162	Ns	A29P		
**714**	7733276	-	CCCC	18.75%	-				SAZ_7192	Up
**715**	7733445	T	G	3.98%	-				SAZ_7193	Dn
**716**	7757697	G	A	3.65%	-	SAZ_7219	Ns	E506K		
**717**	7815056	G	C	8.88%	10.61%				SAZ_7268,SAZ_7267	Up, Dn
**718**	7815060	G	C	7.65%	9.09%				SAZ_7268,SAZ_7267	Up, Dn
**719**	7815065	G	C	4.23%	6.25%				SAZ_7268,SAZ_7267	Up, Dn
**720**	7815068	G	C	-	4.73%				SAZ_7268,SAZ_7267	Up, Dn
**721**	7815740	A	C	-	3.52%	SAZ_7268	Ns	I200L		
**722**	7819445	C	-	3.03%	-				SAZ_7272,SAZ_7271	Up, Dn
**723**	7821979	A	C	3.15%	-				SAZ_7274,SAZ_7273	Up, Dn
**724**	7821982	A	C	3.59%	-				SAZ_7274,SAZ_7273	Up, Dn
**725**	7823055	G	C	17.78%	18.07%	SAZ_7275	Ns	A40P		
**726**	7823058	G	C	6.77%	12.24%	SAZ_7275	Ns	A41P		
**727**	7848627	C	G	5.67%	6.01%	SAZ_7297	Ns	A46P		
**728**	7862845	A	G	100%	100%	SAZ_7317	S	S142S		

**729**	7951597	A	C	5.51%	9.52%				SAZ_7400	Up
**730**	7964488	A	C	-	3.14%	SAZ_7413	Ns	T191P		
**731**	7973902	G	A	-	4.83%	SAZ_7420	S	A238A		
**732**	8009485	C	G	44.97%	40.62%	SAZ_7452	Ns	P275R		
**733**	8032717	T	G	-	3.47%	SAZ_7475	Ns	V144G		
**734**	8038102	T	G	-	3.08%				SAZ_7480,SAZ_7479	Up, Dn
**735**	8068333	G	C	11.84%	-	SAZ_7509	Ns	G10R		
**736**	8094631	A	G	7.88%	6.80%				SAZ_7531,SAZ_7532	Up, Dn
**737**	8209341	G	A	9.55%	13.07%	SAZ_7637	S	L57L		
**738**	8262875	T	G	5.66%	-	SAZ_7687	Ns	H430P		
**739**	8288039	C	G	5.19%	-	SAZ_7711	Ns	A11P		
**740**	8288043	C	G	6.92%	-	SAZ_7711	S	P9P		
**741**	8331307	A	C	-	4.23%	SAZ_7753	Ns	H24P		
**742**	8332943	G	C	9.60%	-	SAZ_7754	Ns	A189P		
**743**	8332947	G	C	-	7.34%	SAZ_7754	Ns	R190P		
**744**	8332950	A	C	5.07%	-	SAZ_7754	Ns	H191P		
**745**	8332953	G	C	6.83%	7.75%	SAZ_7754	Ns	R192P		
**746**	8344595	G	C	7.45%	6.67%	SAZ_7768	Ns	A294P		
**747**	8349398	C	G	3.59%	-	SAZ_7774	S	P71P		
**748**	8381139	A	G	100%	-				SAZ_7805,SAZ_7806,SAZ_7807	Up
**749**	8381144	A	C	100%	-	SAZ_7807	Ns	M1L		
**750**	8381154	G	T	100%	-	SAZ_7807	Ns	R4L		
**751**	8381158	G	C	100%	100%	SAZ_7807	S	A5A		
**752**	8381167	G	A	96.81%	100%	SAZ_7807	S	E8E		
**753**	8381184	G	T	100%	98.73%	SAZ_7807	Ns	R14L		
**754**	8381200	C	T	98.36%	100%	SAZ_7807	S	G19G		
**755**	8386430	G	C	-	4.61%				SAZ_7811,SAZ_7812	Up, Dn
**756**	8391463	C	G	13.09%	14.39%				SAZ_7815,SAZ_7817,SAZ_7816	Up, Dn
**757**	8391467	C	G	20.86%	24.51%				SAZ_7815,SAZ_7817,SAZ_7816	Up, Dn
**758**	8407393	G	C	10.32%	-	SAZ_7833	Ns	R149P		
**759**	8407446	G	C	-	4.96%	SAZ_7833	Ns	A167P		
**760**	8414831	T	G	4.32%	-	SAZ_7840	Ns	T43P		
**761**	8415233	T	G	-	3.32%				SAZ_7840,SAZ_7841	Up
**762**	8496014	C	G	5.69%	-				SAZ_7920,SAZ_7921	Up, Dn
**763**	8570218	C	G	6.57%	6.47%	SAZ_8016	Ns	A321G		

**764**	8570439	-	C	94.64%	-				SAZ_8016,SAZ_8017	Dn
**765**	8570455	A	G	98.61%	100%				SAZ_8016,SAZ_8017	Dn
**766**	8570471	C	A	98.63%	100%				SAZ_8016,SAZ_8017	Dn
**767**	8570487	C	G	100%	100%				SAZ_8016,SAZ_8017	Dn
**768**	8570507	A	G	100%	-				SAZ_8016,SAZ_8017	Dn
**769**	8570520	A	G	98.08%	-				SAZ_8016,SAZ_8017	Dn
**770**	8570668	G	T	100%	-	SAZ_8017	Ns	A237E		
**771**	8570686	C	A	99.03%	100%	SAZ_8017	Ns	R231L		
**772**	8574295	T	G	-	3.68%				SAZ_8020	Dn
**773**	8684385	A	C	-	3.03%	SAZ_8129	Ns	H185P		
**774**	8686047	C	G	4.29%	-	SAZ_8131	Ns	R365P		
**775**	8694246	A	C	-	3.98%	SAZ_8139	Ns	T118P		
**776**	8697396	-	CC	17.61%	8.53%				SAZ_8142,SAZ_8143	Dn
**777**	8697398	-	TCCC	17.01%	8.27%				SAZ_8142,SAZ_8143	Dn
**778**	8697402	G	C	8.82%	7.96%				SAZ_8142,SAZ_8143	Dn
**779**	8697405	G	C	10.75%	10.38%				SAZ_8142,SAZ_8143	Dn
**780**	8697412	-	CGGCGGGCA	4.91%	5.59%				SAZ_8142,SAZ_8143	Dn
**781**	8697413	-	GG	8.53%	11.11%				SAZ_8142,SAZ_8143	Dn
**782**	8723327	C	G	-	6.11%	SAZ_8166	S	P48P		
**783**	8743120	G	C	13.51%	-				SAZ_8183,SAZ_8181,SAZ_8182	Up, Dn
**784**	8763000	T	G	-	3.12%	SAZ_8203,SAZ_8204	Ns	H4P		
**785**	8772597	G	C	5.03%	-				SAZ_8212,SAZ_8213	Up
**786**	8789182	T	G	4.12%	-	SAZ_8231	Ns	T305P		
**787**	8821880	G	T	98.29%	100%	SAZ_8261	Ns	G78V		
**788**	8821915	G	C	97.92%	100%	SAZ_8261	Ns	V90L		
**789**	8821927	G	T	100%	100%	SAZ_8261	Ns	D94Y		
**790**	8830881	A	C	3.27%	-	SAZ_8270	Ns	V371G		
**791**	8856149	G	A	-	8.60%	SAZ_8294	Ns	E463K		
**792**	8878541	G	C	44.07%	31.41%	SAZ_8320	S	S422S		
**793**	8880867	C	A	100%	-	SAZ_8322	Ns	R203M		
**794**	8972523	T	G	-	3.68%	SAZ_8414	Ns	V317G		
**795**	9038879	T	G	-	3.17%	SAZ_8482	Ns	E1987A		
**796**	9061489	G	C	14.72%	19%	SAZ_8496	Ns	A220G		
**797**	9061492	A	C	6.71%	9.30%	SAZ_8496	Ns	V219G		
**798**	9061495	G	C	9.25%	11.70%	SAZ_8496	Ns	A218G		

**799**	9073008	G	C	-	7.69%				SAZ_8506,SAZ_8507	Dn
**800**	9089878	T	G	-	3.21%	SAZ_8527	Ns	D230A		
**801**	9096894	A	G	32.80%	31%	SAZ_8534	S	N247N		
**802**	9136001	G	T	99.18%	100%	SAZ_8575	Ns	T226N		
**803**	9136040	C	A	99.38%	99.24%	SAZ_8575	Ns	G213V		
**804**	9155131	T	G	6.21%	5.83%				SAZ_8591,SAZ_8590	Up, Dn
**805**	9176767	A	C	3.11%	-	SAZ_8616	Ns	T364P		
**806**	9213077	A	C	3.06%	-	SAZ_8649	Ns	T10P		
**807**	9246026	G	A	100%	-	SAZ_8654	S	Q749Q		
**808**	9260196	CCC	-	38%	-	SAZ_8655	nonFS	3676_3677del		
**809**	9267137	G	C	9.62%	-				SAZ_8657,SAZ_8656	Up, Dn
**810**	9267142	G	C	6.38%	-				SAZ_8657,SAZ_8656	Up, Dn
**811**	9274035	G	C	7.44%	-	SAZ_8662	S	P432P		
**812**	9274051	G	C	8.77%	-	SAZ_8662	Ns	A438P		
**813**	9297984	C	G	100%	100%	SAZ_8665	Ns	L996V		
**814**	9299495	A	G	98.86%	100%	SAZ_8665	S	R1499R		
**815**	9302503	A	C	5.41%	6.38%	SAZ_8666	S	T568T		
**816**	9302512	C	T	8.21%	8.87%	SAZ_8666	S	V571V		
**817**	9302521	T	G	9.56%	14.08%	SAZ_8666	S	G574G		
**818**	9302725	C	T	28.36%	35.43%	SAZ_8666	S	D642D		
**819**	9302842	C	T	18.99%	18.62%	SAZ_8666	S	D681D		
**820**	9321838	T	C	100%	100%	SAZ_8671	S	R781R		
**821**	9321889	G	C	100%	100%	SAZ_8671	S	G798G		
**822**	9322021	T	G	100%	98.31%	SAZ_8671	Ns	F842L		
**823**	9322552	C	T	98.69%	99.35%	SAZ_8671	S	G1019G		
**824**	9322564	C	T	100%	100%	SAZ_8671	S	N1023N		
**825**	9353554	A	G	100%	100%	SAZ_8691	S	A790A		
**826**	9398102	-	G	32.94%	33.85%				SAZ_8729,SAZ_8730	Up, Dn
**827**	9398881	G	T	3.27%	-				SAZ_8730,SAZ_8731	IR
**828**	9402235	A	C	3.65%	-				SAZ_8734,SAZ_8735	Up
**829**	9405784	T	G	3.32%	-	SAZ_8738	Ns	H481P		
**830**	9407177	C	G	5.19%	-	SAZ_8738	Ns	A17P		
**831**	9409320	C	A	100%	-	SAZ_8741	Ns	G397V		
**832**	9421627	G	-	14.02%	17.33%				SAZ_8748,SAZ_8749	Up, Dn
**833**	9470272	-	GTGGGCATG	21.15%	-	SAZ_8790	nonFS	E1384delinsEVGM		

**834**	9563086	G	A	10.47%	-				SAZ_8883	Dn
**835**	9639547	G	C	12.07%	17.95%	SAZ_8963	S	P1766P		
**836**	9639550	A	C	19.79%	10.77%	SAZ_8963	S	P1767P		
**837**	9639554	T	C	6.25%	-	SAZ_8963	Ns	S1769P		
**838**	9639566	G	C	5.22%	-	SAZ_8963	Ns	A1773P		

*Abbreviations: Ref: genome reference sequence; Ns: non-synonymous mutaion; S: synonymous mutation; FS: frame shift muation; Up: Distances within 300 bp upstream of the coding regions of genes; Dn: Distances within 300 bp downstream of the coding regions of genes; IR: intergenenic region , distance beyond 300 bp upstream or downstream of the coding regions of the nearest genes.

Table S5 Genes associated with PL yield

Gene	low-specific	high-specific	COG	Molecular function
SAZ_0249		Y72S		hypothetical protein
SAZ_0323	H259P		[R]	alpha/beta hydrolase fold protein
SAZ_0379	H289P		[C]	oxidoreductase
SAZ_0496	T258P		[S]	AF110772_1 unknown
SAZ_0569	H275P		[M]	GouI
SAZ_0612	H214P			hypothetical protein
SAZ_0692	R316H		[R]	ABC transporter
SAZ_0870		S15R	[C]	FAD-binding monooxygenase protein
SAZ_0877		Q272P	[K]	MerR family transcriptional regulator
SAZ_0972	T13P		[C]	flavohemoprotein
SAZ_1010		G590D	[G]	glycoside hydrolase 15-like protein
SAZ_1011	V529G	V621I	[P]	potassium-transporting ATPase subunit B

SAZ_1016	T307P			amino acid transporter
SAZ_1062		T527P	[P]	Mn2+ or Fe2+ transporter
SAZ_1118		V1147G	[Q]	putative NRPS-type-I PKS fusion protein
SAZ_1165		T180P	[M]	hypothetical protein
SAZ_1195	N8T		[S]	hypothetical protein
SAZ_1227		T57P		hypothetical protein
SAZ_1229		N357H	[C]	citrate synthase
SAZ_1284		V116G		hypothetical protein
SAZ_1389		F331C	[K]	hypothetical protein
SAZ_1419	T385P		[T]	two-component sensor kinase
SAZ_1473		T117P	[S]	putative surface layer protein
SAZ_1530		T357P	[T]	magnesium or manganese-dependent protein phosphatase
SAZ_1564	P103S		[F]	hypothetical protein
SAZ_1614	T103P		[E]	cholesterol oxidase
SAZ_1645		V37G		hypothetical protein
SAZ_1669	A164P			hypothetical protein
SAZ_1718		T134P	[K]	acetyltransferase
SAZ_1760		V297G	[M]	glycosyl transferase family protein

SAZ_1831		T1140fs	[T]	hypothetical protein
SAZ_1881	H207P		[K]	TetR-family transcriptional regulator
SAZ_1981		V85G	[I]	acyl-CoA dehydrogenase
SAZ_2021	A181G		[K]	LysR family transcriptional regulator
SAZ_2192	A106G		[F]	guanylate kinase
SAZ_2193	T7N			hypothetical protein
SAZ_2269		R71fs		nicotinate-nucleotide-dimethylbenzimidazole phosphoribosyltransferase, partial
SAZ_2281		T445P	[G]	major facilitator superfamily
SAZ_2395		T542P	[G]	ABC transporter
SAZ_2443		V33G	[L]	CRISPR-associated protein
SAZ_2574	V258G		[G]	HAD-superfamily hydrolase, subfamily IIA
SAZ_2595		L53P	[K]	two-component system response regulator
SAZ_2597	S8P			aminoglycoside 2'-N-acetyltransferase
SAZ_2625	A756P, T753P, H749P		[C]	acyl-CoA synthetase
SAZ_2647	T352P			hypothetical protein SU9_05461, partial
SAZ_2816		T97P	[C]	aldehyde dehydrogenase
SAZ_2908	A42P		[R]	hypothetical protein

SAZ_2922	A7P		[E]	branched-chain amino acid ABC transporter ATP-binding protein
SAZ_2928		S89R	[S]	hypothetical protein
SAZ_2929	V26F		[F]	5'-nucleotidase
SAZ_3031	A99G			hypothetical protein
SAZ_3141		F119C, T13P	[R]	hypothetical protein
SAZ_3160	A6P	V303G	[R]	amidohydrolase
SAZ_3190		L345R	[G]	major facilitator superfamily protein
SAZ_3299	T680P		[G]	multidrug-efflux transporter
SAZ_3304		V488G		hypothetical protein
SAZ_3318	H42P			hypothetical protein
SAZ_3328		H202P	[T]	stress protein
SAZ_3363	V213G		[G]	ribose ABC transporter substrate-binding protein
SAZ_3378	T874P	T663P		hypothetical protein
SAZ_3413		T180P		hypothetical protein
SAZ_3418	V174G		[R]	metallopeptidase
SAZ_3494	T67fs		[K]	heat-inducible transcription repressor
SAZ_3587		E19A		hypothetical protein

SAZ_3618	A147G		[R]	ATP-binding membrane protein, partial
SAZ_3850		L333V	[G]	ABC transporter permease, partial
SAZ_3853		N45T		hypothetical protein
SAZ_3863	W363G		[R]	hydrolase
SAZ_3874		P1341T	[E]	NAD-glutamate dehydrogenase
SAZ_3986	P87T, P90T, P90Q		[V]	ABC transporter ATP-binding protein
SAZ_4016	W112R	V69I		hypothetical protein
SAZ_4102		T623A	[R]	penicillin acylase
SAZ_4103		T420P	[P]	potassium/proton antiporter
SAZ_4111		V364G	[G]	major facilitator superfamily protein
SAZ_4132	A132V		[K]	RNA polymerase principal sigma factor hrdD
SAZ_4218		W144G	[K]	ECF subfamily RNA polymerase sigma factor
SAZ_4270	M1R		[K]	DNA-directed RNA polymerase subunit alpha
SAZ_4432		T21P	[H]	delta-aminolevulinic acid dehydratase
SAZ_4528		A882P	[O]	ATP-dependent chaperone ClpB
SAZ_4534		G205A	[O]	heat shock protein GrpE
SAZ_4571		T578P	[E]	amino acid permease-associated region
SAZ_4578	Y40D		[L]	conserved hypothetical protein

SAZ_4715		T238P	[M]	sortase
SAZ_4718	A63P		[M]	hypothetical protein
SAZ_4911	G188W		[G]	major facilitator superfamily permease
SAZ_5014	V294G		[V]	beta-lactamase
SAZ_5199		E152K		hypothetical protein
SAZ_5290	V144G		[J]	HemK family modification methylase
SAZ_5477	P501L	G73V	[C]	FAD linked oxidase domain-containing protein
SAZ_5482	V244M			hypothetical protein
SAZ_5548		A18P	[V]	hypothetical protein
SAZ_5549		Y142S	[K]	hypothetical protein
SAZ_5643		H296P		hypothetical protein
SAZ_5699		R20P		OHCU decarboxylase
SAZ_5716		T429P	[C]	hypothetical protein
SAZ_5787		Y89D		hypothetical protein
SAZ_5788		V1G		RNA polymerase ECF-subfamily sigma factor
SAZ_5826	A188P			secreted protein
SAZ_6010	V285G		[P]	KatA1
SAZ_6013	V35G			TetR family transcriptional regulator

SAZ_6022		V399G	[T]	hypothetical protein
SAZ_6026		Y13S	[M]	putative penicillin-binding protein
SAZ_6066		T41P		peptidase M7 snapalysin
SAZ_6141	V948fs		[Q]	beta-ketoacyl synthase
SAZ_6166		Y126S	[L]	hypothetical protein (sigma-70 factor)
SAZ_6197		T59P	[K]	TetR-family transcriptional regulator
SAZ_6202	T88fs,		[G]	pyruvate kinase
SAZ_6320	T655P, T658P			lyase
SAZ_6414		T211P	[M]	M23 family peptidase
SAZ_6435		T183P	[C]	aldehyde dehydrogenase
SAZ_6481	L270P		[D]	hypothetical protein
SAZ_6525		A282P	[V]	ABC-type drug export system, ATP-binding protein
SAZ_6560		T786P	[Q]	amino acid adenylation domain-containing protein, partial
SAZ_6565		L23P	[Q]	hypothetical protein
SAZ_6619	A35P		[R]	protease
SAZ_6624	V210G		[E]	LysE family efflux protein
SAZ_6654		D241fs	[G]	glycogen debranching protein
SAZ_6691		L78V		hypothetical protein

SAZ_6695	S328A, A329fs		[S]	ribonuclease BN
SAZ_6739	W210X		[K]	MerR family transcriptional regulator
SAZ_6762		L78R	[R]	hypothetical protein
SAZ_6873	R43C		[R]	short-chain dehydrogenase/reductase SDR
SAZ_6911		L408F		agarase
SAZ_6912		A352T		hypothetical protein
SAZ_6927	T189P			hypothetical protein
SAZ_6952	T63P			hypothetical protein
SAZ_7056	T158P			hypothetical protein
SAZ_7134		D314A	[R]	methyltransferase
SAZ_7162	A29P			hypothetical protein
SAZ_7219	E506K		[S]	SNF2/RAD54 family helicase
SAZ_7268		I200L	[G]	AF164960_3 transporter
SAZ_7413		T191P	[C]	fatty acid-CoA racemase
SAZ_7475		V144G		hypothetical protein
SAZ_7509	G10R		[R]	amidohydrolase
SAZ_7687	H430P		[M]	cyclopropane-fatty-acyl-phospholipid synthase
SAZ_7711	A11P		[R]	putative sugar acetyltransferase

SAZ_7753		H24P	[E]	pyridoxal-5'-phosphate-dependent protein beta subunit
SAZ_7754	A189P, H191P	R190P	[G]	putative MFS transporter
SAZ_7807	M1L, R4L		[M]	apolipoprotein N-acyltransferase
SAZ_7833	R149P	A167P	[K]	LacI family transcriptional regulator
SAZ_7840	T43P		[R]	hypothetical protein
SAZ_8017	A237E		[L]	hypothetical protein
SAZ_8129		H185P	[I]	acetyl-CoA acetyltransferase
SAZ_8131	R365P		[R]	enoyl-ACP reductase II
SAZ_8139		T118P	[E]	4-hydroxy-2-oxovalerate aldolase
SAZ_8203		H4P		hypothetical protein
SAZ_8204		T431P	[R]	von Willebrand factor type A
SAZ_8231	T305P			putative cytoplasmic protein
SAZ_8270	V371G			hypothetical protein
SAZ_8294		E463K	[J]	amidase
SAZ_8322	R203M		[E]	methionine synthase
SAZ_8414		V317G		VanZ-like protein
SAZ_8482		E1987A	[G]	Rhs protein, partial
SAZ_8527		D230A		membrane protein, PF10067 family

SAZ_8616	T364P		[I]	acyl-CoA dehydrogenase
SAZ_8649	T10P		[M]	AF263912_4NysDIII
SAZ_8655	3676_3677del		[Q]	AF263912_6NysJ
SAZ_8662	A438P		[Q]	AF263912_13NysA
SAZ_8738	H481P, A17P		[V]	beta-lactamase
SAZ_8741	G397V		[C]	radical SAM domain containing enzyme
SAZ_8790	E1384delinsEVGM		[Q]	beta-ketoacyl synthase
SAZ_8963	S1769P, A1773P		[Q]	type I polyketide synthase
